# Multi-omics analysis identifies fibroblast IGFBP5 as a key target of anti-TNFα therapy in ulcerative colitis

**DOI:** 10.3389/fimmu.2026.1841149

**Published:** 2026-08-20

**Authors:** Peihong Li, Yikun Zhang, Liuqin Zhao, Yiwen Wang, Hongyi Hu, Siqing Guo, Tingting Zhang, Zhancheng Lin, Jiang Lin, Kexin Zeng, Linda Zhong, Changqin Liu, Boyun Sun

**Affiliations:** 1 Department of Gastroenterology, Longhua Hospital, Shanghai University of Traditional Chinese Medicine, Shanghai, China; 2 School of Biological Sciences, Nanyang Technological University, Singapore, Singapore; 3 Fengxian District Hospital of Traditional Chinese Medicine, Shanghai, China; 4 Department of Internal Medicine, Tianshan Hospital of Traditional Chinese Medicine, Shanghai, China; 5 Department of Coloproctology, Longhua Hospital, Shanghai University of Traditional Chinese Medicine, Shanghai, China; 6 Department of Colorectal Surgery, Shanghai Yangpu Hospital of Traditional Chinese Medicine, Shanghai, China; 7 Department of Critical Care Medicine, Longhua Hospital, Shanghai University of Traditional Chinese Medicine, Shanghai, China; 8 College of Plant Protection, Hunan Agricultural University, Changsha, China; 9 Department of Gastroenterology, Shanghai Tenth People’s Hospital, Shanghai, China

**Keywords:** anti-TNFα resistance, fibroblasts, IGFBP5, scRNA-seq, spatial transcriptomics, ulcerative colitis

## Abstract

**Background:**

Resistance to anti-TNFα therapy (e.g., infliximab) in ulcerative colitis (UC) remains a significant clinical challenge, with the underlying cellular and spatial mechanisms poorly understood.

**Methods:**

We integrated bulk transcriptomics, single-cell RNA sequencing (scRNA-seq), and spatial transcriptomics (ST) from clinical cohorts to systematically map the cellular landscape and identify key determinants of treatment response, followed by validation in independent clinical cohorts and *in vitro* functional experiments.

**Results:**

Multi-omics cross-analysis pinpointed insulin-like growth factor-binding protein 5 (IGFBP5) as a fibroblast-specific, spatially enriched gene strongly associated with infliximab non-response. Functional analyses linked IGFBP5 to inflammatory pathways and a distinct immune-activated microenvironment. Cell-cell communication analysis revealed that IGFBP5-high fibroblasts exhibit enhanced crosstalk with monocytes, endothelial cells, and T cells via VEGF, MIF, and WNT signaling. Validation in clinical samples and functional experiments confirmed elevated expression of IGFBP5 and its downstream effectors EGR1 and VEGFR2 in non-responders, Mechanistically, IGFBP5 promoted activation of the VEGFR2/EGR1 signaling axis and enhanced the secretion of inflammatory mediators, including IL-6, CXCL12, and CCL2, thereby contributing to a pro-inflammatory fibroblast phenotype.

**Conclusions:**

This study establishes fibroblast-derived IGFBP5 as a central mediator of anti-TNFα resistance in UC. The IGFBP5-EGR1-VEGFR2 axis may represent a candidate predictive biomarker and a promising target for overcoming treatment resistance.

## 1 Introduction

Ulcerative colitis (UC) is a chronic, idiopathic inflammatory disorder affecting the colon, with a rising incidence worldwide. Its pathogenesis involves a complex interplay of genetic susceptibility, epithelial barrier defects, dysregulated immune responses, and environmental factors (1). Immune-mediated inflammatory cascades ultimately result in intestinal tissue damage, which can lead to colonic perforation, hemorrhage, and, in some cases, malignant transformation. Patients with moderate-to-severe active UC often require anti-tumor necrosis factor (TNF) antibody therapy (2). In patients with IBD treated with anti-TNFα-α biologics, a substantial proportion fail to achieve an initial clinical response, with up to one-third of patients exhibiting primary nonresponse, and a significant fraction of initial responders subsequently experience loss of response during maintenance therapy (3). Thus, elucidating the underlying immune-mediated pathogenic mechanisms of UC is critical for guiding the development of novel therapeutic strategies in future clinical trials.

Single-cell RNA sequencing (scRNA-seq) is a high-throughput technology that can resolve gene expression at the level of individual cells. It has emerged as a powerful tool for analyzing cellular heterogeneity and the intestinal immune system (4). Using scRNA-seq, researchers can identify the distinct transcriptional profiles of immune and non-immune cell populations, including fibroblasts and intestinal epithelial cells, in diseased tissues. This helps them understand the roles these cells play in disease initiation and progression (5, 6). Meanwhile, spatial transcriptomics (ST) has advanced and matured. This new approach has significant advantages. It preserves tissue spatial information, interrogates the microenvironment, and resolves spatial heterogeneity. ST complements single-cell analyses. It provides a more comprehensive view of tissue architecture and cellular function (7, 8). Integrating ST enables precise mapping of diverse cell populations in tissue contexts. It also characterizes their intercellular interactions. This is critical for understanding alterations in the intestinal microenvironment (9). Through ST, immune cells, epithelial cells, and fibroblasts can be localized within gut tissue, offering insights into their coordinated or reciprocal interactions (10, 11). This combined approach enhances our understanding of the mechanisms underlying treatment resistance and provides a foundation for developing personalized therapeutic strategies.

Fibroblasts, a type of mesenchymal cell, play a crucial role in maintaining intestinal tissue homeostasis, mediating immune responses, and driving tissue remodeling. Upon exposure to microbial stimuli or tissue injury, innate immune cells such as macrophages and dendritic cells trigger inflammatory cascades, which in turn promote fibroblast activation and the release of key mediators, including TGF-β, TNFα, and interleukins (12). Interactions between fibroblasts and other cell types represent a critical mechanism underlying therapeutic resistance. For instance, neutrophil-dependent IL-1 signaling may facilitate the activation of IL-1R+ fibroblasts, contributing to resistance to biologic therapies (13). In UC, inflammation-associated fibroblasts (IAFs) expand markedly within inflamed tissues, participating in both inflammatory responses and tissue remodeling, and acting as central nodes in colitis-associated cellular interaction networks (14). Resistance to anti-TNFα therapy has been linked to the enrichment of OSM and its receptor OSMR in IAFs, which, when expressed in immune cells, promotes the development of resistant phenotypes. During anti-TNFα treatment, IAFs and inflammatory monocytes may engage in compensatory mechanisms, either synergistically or redundantly enhancing OSM and TNF signaling to activate downstream targets, thereby sustaining or amplifying inflammation and ultimately driving resistance (15). In UC patients, anti-TNFα-α therapy was associated with a marked reduction in LAMP3⁺ cDC3s in responders, accompanied by diminished ligand-receptor interactions between cDC3s, fibroblasts, and epithelial cells, whereas non-responders exhibited no significant changes in cDC3 abundance or intercellular communication (16). Collectively, these observations highlight that fibroblast-immune cell interactions and downstream compensatory mechanisms in UC represent potential contributors to anti-TNFα resistance.

**Figure 1:**
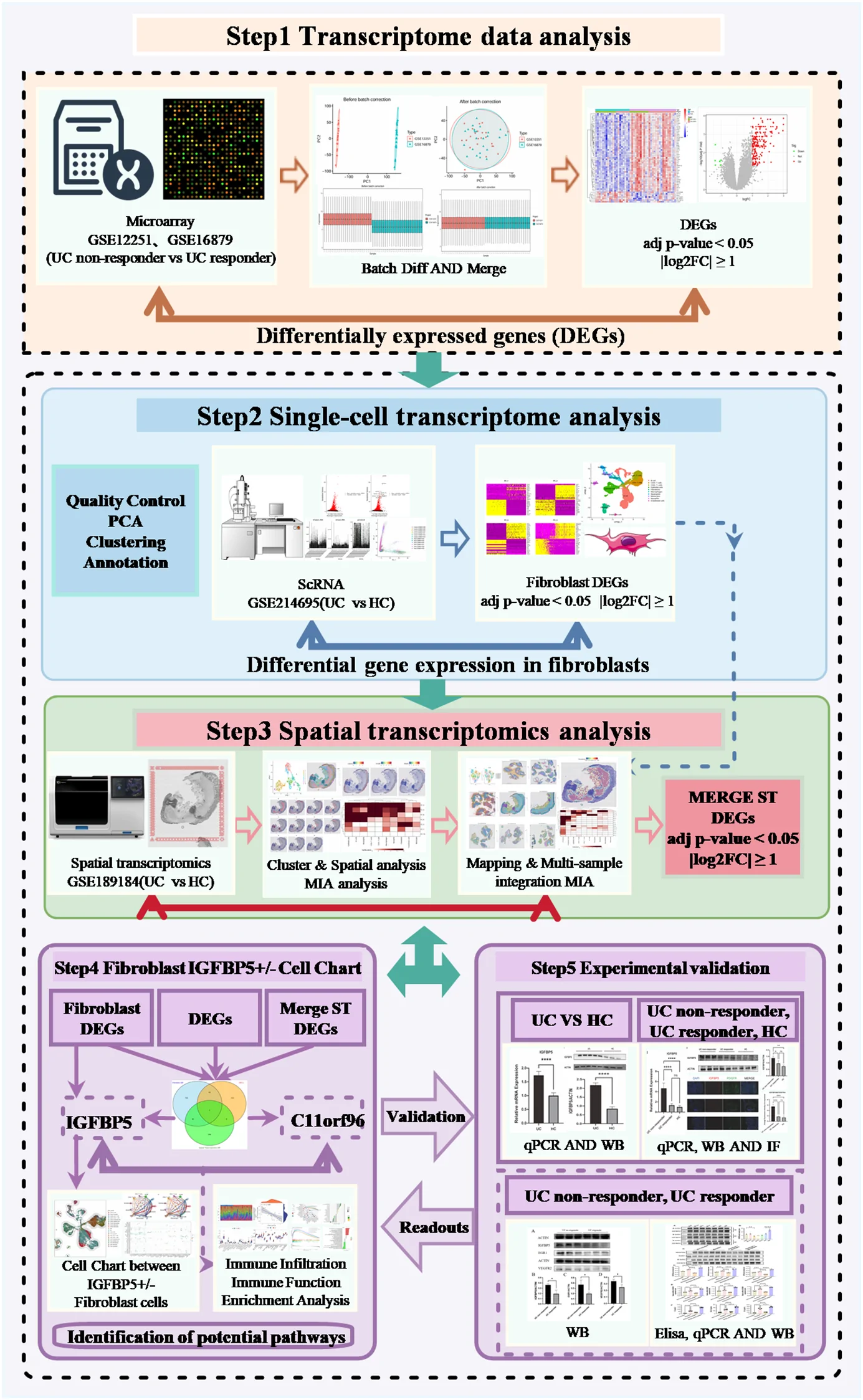
The overall flowchart of this research.

In this study, by integrating bulk transcriptomics, scRNA-seq, and ST, we focused on UC-associated fibroblasts to investigate the potential mechanisms through which key genes may contribute to infliximab resistance, providing novel insights for therapeutic strategies against treatment-refractory disease. The detailed analytic workflow was shown in **Figure 1**.

## 2 Materials and methods

### 2.1 Data collection

Data for this study were collected from publicly available gene expression databases (https://www.ncbi.nlm.nih.gov/geo/). The bulk transcriptome data, including datasets GSE12251 and GSE16879, were integrated to identify differentially expressed genes (DEGs) between infliximab responders and non-responders. An independent dataset, GSE73661, was used for subsequent validation of key candidate genes. Treatment response and non-response were defined according to the original study criteria for each public dataset. For GSE12251, response to infliximab was defined as complete mucosal healing, with a Mayo endoscopic subscore (MES) of 0 or 1 and a histological score of 0 or 1, assessed 8 weeks after induction therapy. For GSE16879, response was evaluated 4–6 weeks after the first infliximab infusion and was defined as complete mucosal healing, corresponding to an MES of 0 or 1 and a histological score of 0 or 1. For the infliximab subset of GSE73661, response was defined as endoscopic mucosal healing, corresponding to an MES of 0 or 1, assessed 4–6 weeks after induction therapy. Accordingly, patients classified as non-responders represented primary non-response (PNR). To resolve cellular heterogeneity, scRNA-Seq data from the GSE214695 dataset, which encompasses colonic tissues from six UC patients and six healthy controls (HC), were analyzed. Furthermore, to investigate the transcriptional landscape within its native tissue architecture, ST data from the GSE189184 dataset, comprising six UC samples and two HC, were incorporated. The integration of scRNA-seq and ST data enabled high-precision spatial mapping of cell types. All included public datasets were originally approved by their respective ethical review committees. The basic information of the datasets is detailed in **Table 1**.

**Table 1:** Basic information of the datasets included in this study.

Dataset	Type	Platform	Sample	Biopsy source	Response definition
GSE12251	Microarray	GPL570	UC non-responder (n = 11); UC responder (n = 12)	Colon tissue	Complete mucosal healing (MES 0-1 + histological score 0-1), Week 8
GSE16879	Microarray	GPL570	UC non-responder (n = 16); UC responder (n = 8)	Colon tissue	Complete mucosal healing (MES 0-1 + histological score 0-1), Week 4-6
GSE73661	Microarray	GPL6244	UC non-responder (n = 15); UC responder (n = 8)	Colon tissue	Endoscopic mucosal healing (MES 0-1), Week 4-6
GSE214695	scRNA-seq	GPL18573	UC (n = 6); HC (n = 6)	Colon tissue	/
GSE189184	Spatial transcriptomics	GPL18573	UC (n = 7); HC (n = 2)	Colon tissue	/

### 2.2 GEO data preprocessing and DEGs analysis

To identify transcriptional signatures associated with infliximab response at the bulk tissue level, we integrated the normalized expression matrices from datasets GSE12251 and GSE16879. The “limma” package was utilized for data normalization, and the ComBat method was applied to correct for batch effects, with the effectiveness of correction confirmed by principal component analysis (PCA). DEGs between infliximab responders and non-responders were defined using an FDR-adjusted p-value < 0.05 and an absolute log2-fold change (|log2FC|) ≥ 1. The results were visualized using volcano plots and heatmaps to depict global transcriptional differences.

### 2.3 ScRNA-seq analysis

To resolve cellular heterogeneity and identify cell type-specific gene expression patterns in UC, we analyzed the scRNA-seq data from GSE214695. Stringent quality control was applied, retaining cells expressing at least 100 genes, genes detected in a minimum of 3 cells, and cells with mitochondrial gene content below 15%. Data normalization was performed using the LogNormalize method in Seurat. The 1,500 most highly variable genes were selected for downstream analysis. Following data scaling, dimensionality reduction was carried out via PCA, and the top 20 principal components were used for cell clustering based on a shared nearest neighbor graph (FindNeighbors) and the FindClusters function. The resulting clusters were visualized in two dimensions using UMAP. To define the transcriptional identity of each cluster, differential expression analysis was performed using the FindAllMarkers function (logFC threshold > 1, minimum expression fraction > 0.25).

### 2.4 Spatial transcriptomics analysis

To interrogate the transcriptional landscape of UC within its native tissue architecture, we processed the ST data from GSE189184. Seurat objects were constructed with integrated spatial coordinates for preliminary visualization. The SCTransform method was employed for normalization and removal of technical noise. Subsequent steps included dimensionality reduction via PCA, construction of a nearest-neighbor graph, and clustering to identify spatially coherent expression domains. These spatial domains were visualized using UMAP embeddings and spatial mapping plots (DimPlot and SpatialDimPlot). Differential gene expression analysis (logFC > 1, FDR-adjusted p-value < 0.05) was conducted to identify marker genes for each spatial domain. Furthermore, Moran’s I statistic was applied to assess spatial autocorrelation and select genes with significant spatial heterogeneity, which were then visualized to reveal the spatial dynamics of key genes within the tissue context.

### 2.5 Integrated analysis of scRNA-seq and ST data

scRNA-seq gives single-cell transcriptomes without location; ST keeps location but misses single-cell detail. Integrating the two yields cell-type maps with both. Briefly, both scRNA-seq and ST datasets were independently normalized using SCTransform and underwent dimensionality reduction (PCA and UMAP). Cell types defined in the scRNA-seq analysis were then annotated onto the ST spots using anchor-based integration (FindTransferAnchors and TransferData functions in Seurat). This allowed for the spatial visualization of cell cluster distribution patterns, revealing the correspondence between cell types and their specific tissue microenvironments.

### 2.6 Multi-omics integration analysis

To investigate the spatial enrichment patterns of specific cell types, we implemented a MIA strategy. This approach integrated the cell type-specific DEGs identified from scRNA-seq with the spatial expression data from ST. Functional enrichment analysis was first performed on the scRNA-seq-derived DEGs for each cell type using the clusterProfiler package. Their significance within the spatial expression landscape was then evaluated. After appropriate data matrix transformation and missing value imputation, heatmaps were generated to depict the spatial enrichment features of distinct cell types, thereby elucidating potential relationships between cellular composition and spatial expression patterns.

### 2.7 Multi-sample integration analysis of ST data

To characterize spatial expression patterns and cellular composition across UC tissues, all spatial transcriptomics (ST) samples were integrated into a unified Seurat object and processed for normalization, dimensionality reduction, clustering, and UMAP visualization. Cluster-specific marker genes were identified using differential expression analysis (logFC > 1, FDR-adjusted p-value < 0.05). Multi-sample multi-omics integration (MIA) combining scRNA-seq and ST data was then performed to assess spatial enrichment of cell types across samples. Heatmaps were generated to visualize spatial distribution patterns and cellular remodeling in UC tissues.

### 2.8 Functional enrichment analysis

To elucidate the biological implications of the spatially resolved DEGs, we performed functional enrichment analysis on key genes identified from the ST data. Gene Ontology (GO) annotation was used to systematically assess their roles in biological processes (BP), molecular functions (MF), and cellular components (CC). Concurrently, Kyoto Encyclopedia of Genes and Genomes (KEGG) pathway analysis was conducted to identify their involvement in higher-order signaling and metabolic networks. The enrichment results were visualized using heatmaps and circos plots to depict the functional distribution and relationships of significant pathways.

### 2.9 Intersection analysis of key candidate genes

To pinpoint the most critical genes linking fibroblast biology to therapeutic response, we performed a systematic intersection analysis. Candidate genes were defined as the overlap between three gene sets (1): fibroblast-specific DEGs derived from scRNA-seq analysis (2), bulk tissue DEGs associated with infliximab response, and (3) genes exhibiting significant spatial expression patterns in the ST data. The expression profiles of these intersecting key genes were subsequently visualized and corroborated across both scRNA-seq and ST modalities. Their differential expression patterns and relevance to treatment response were independently validated using the external dataset GSE73661. Finally, the differential expression patterns of the identified candidate genes were independently validated in three external cohorts treated with different biologic agents (GSE73661, GSE92415, and GSE206285), allowing evaluation of their association with treatment response beyond infliximab therapy. Detailed information on these validation cohorts is summarized in **Supplementary Table 1**.

### 2.10 Analysis of immune infiltration and function

To explore the relationship between key gene expression and the immune microenvironment, immune cell infiltration and functional states were analyzed using bulk transcriptomic data. Relative abundances of 22 immune cell types were estimated using CIBERSORT, and samples with deconvolution p < 0.05 were included for further analysis. Correlations between gene expression and immune cell proportions were assessed using appropriate statistical methods. Immune functional activity was evaluated using ssGSEA based on an immune-related gene set (immune.gmt), and enrichment scores were compared between high- and low-expression groups using the Wilcoxon rank-sum test.

### 2.11 Gene set enrichment and variation analysis

To gain insights into the biological pathways and functional states associated with key gene expression, we used Gene Set Enrichment Analysis (GSEA) and Gene Set Variation Analysis (GSVA). GSEA was used to identify KEGG and GO pathways that were differentially enriched between the high- and low- key gene expression groups. Gene sets containing 15 to 500 genes were evaluated using a weighted enrichment statistic with 1,000 permutations, and significance was defined as a nominal p-value < 0.05 and a false discovery rate (FDR) < 0.05. In parallel, GSVA was applied as an unsupervised method to transform the gene-level expression matrix into a pathway-level score matrix, enabling the assessment of pathway activity variation across individual samples. We analyzed gene sets from the MSigDB collections “h.all.v7.5.1.symbols,” “c2.cp.kegg.v7.5.1.symbols,” and “c2.cp.reactome.v7.5.1.symbols.”

### 2.12 Analysis of fibroblast communication networks

To investigate the influence of key gene expression on intercellular crosstalk, we examined fibroblast-specific communication networks. Using scRNA-seq data, fibroblasts were stratified into IGFBP5-high (IGFBP5-) and IGFBP5-low (IGFBP5-) subpopulations based on the median expression level of IGFBP5 within fibroblasts. Cell-cell communication analysis was performed using CellChat. We restricted the inference to the “Secreted Signaling” subset of the CellChatDB.human database to focus on canonical ligand-receptor interactions. For each subgroup, identical analysis pipelines and parameters were applied to ensure comparability. The analysis involved identification of overexpressed ligands and receptors, inference of ligand-receptor pairing probabilities, and computation of communication probabilities between cell types. A minimum cell number threshold (min.cells=10) was applied during filtering to reduce spurious interactions. Pathway-level communication probabilities were computed using computeCommunProbPathway, followed by network aggregation using aggregateNet. The resulting communication networks were visualized as global interaction maps, group-specific outgoing and incoming signaling patterns, and pathway-level bubble plots. Communication matrices were exported for downstream analysis and interpretation.

### 2.13 Cell culture and maintenance of CCD-18Co fibroblasts

Human colonic fibroblast CCD-18Co cells (GNHu59; Chinese Academy of Sciences Cell Bank) were cultured in Minimum Essential Medium (MEM, Gibco) supplemented with 10% fetal bovine serum (FBS, Gibco) and 1% penicillin-streptomycin (Gibco). Cells were maintained at 37 °C in a humidified incubator with 5% CO₂. Cells between passages 4 and 10 were used for all experiments.

### 2.14 Patient serum collection and preparation

Peripheral blood and colonic tissue samples were collected from patients at Shanghai Tenth People’s Hospital after written informed consent was obtained from all participants. The study protocol was approved by the Ethics Committee of Shanghai Tenth People’s Hospital, Tongji University School of Medicine (Approval No. 23KT87). All patients received standard infliximab induction therapy (Weeks 0, 2, and 6). Treatment response was assessed at Week 14 according to the institutional clinical protocol. Response was defined as endoscopic mucosal healing, corresponding to a MES of 0 or 1. Patients who failed to achieve endoscopic mucosal healing were classified as PNR. Patients with secondary loss of response LOR were not included. Serum was obtained by allowing whole blood to clot at room temperature for 30 min, followed by centrifugation at 3000 × g for 10 min at 4 °C. Supernatants were collected, aliquoted, and stored at -80 °C. To minimize inter-individual variability, sera from individuals within the same group were pooled at equal volumes prior to use. Serum was heat-inactivated at 56 °C for 30 min before cell stimulation to inactivate complement activity. Control cells were maintained in standard medium containing 10% FBS without serum stimulation.

### 2.15 Experimental grouping, transfection, and *in vitro* stimulation model

To investigate the functional role of IGFBP5 under inflammatory conditions, CCD-18Co cells were exposed to serum derived from infliximab-responsive or infliximab-non-responding UC patients. Cells were divided into four experimental groups: Fibroblasts treated with serum from infliximab-responsive UC patients (non-resistant serum group); Fibroblasts treated with serum from infliximab-non-responding UC patients (resistant serum group); Resistant serum-treated fibroblasts transfected with IGFBP5-targeting siRNAs (resistant + si-IGFBP5 group); Resistant serum-treated fibroblasts transfected with IGFBP5 overexpression plasmid (resistant + OE-IGFBP5 group). Three independent siRNAs (si-1, si-2, si-3) and a negative control siRNA (si-NC) were used for knockdown experiments, and the most efficient siRNA was selected for subsequent assays. For overexpression experiments, a human IGFBP5 expression plasmid (NM_000599.4) and empty vector control were transfected. Following transfection, cells were further stimulated with the corresponding serum for 24 h before sample collection. Transfection efficiency was validated by Western blotting. All experiments were performed in triplicate. Protein extraction and Western blot analysis were performed using standard protocols (RIPA lysis, BCA quantification, SDS-PAGE, PVDF transfer, ECL detection). Primary antibodies included IGFBP5, EGR1, VEGFR2, and β-actin, and band intensities were quantified using ImageJ and normalized to β-actin.

### 2.16 Quantitative PCR validation

To experimentally validate the mRNA expression levels of key candidates, quantitative reverse transcription PCR (qPCR) was performed on human colonic tissue samples. The cohort for two-group comparisons included 10 UC patients and 10 HC, while the cohort for three-group comparisons comprised 9 infliximab non-responders, 9 infliximab responders, and 10 HC. Total RNA was extracted from colonic tissues using TRIzol reagent (Thermo Fisher Scientific, Carlsbad, CA, USA) according to the manufacturer’s instructions. RNA concentration and purity were assessed using a UV spectrophotometer (Eppendorf, Germany). Reverse transcription was performed with Evo M-MLVRT Master Mix (Accurate Biotechnology, AG11706, Changsha, China), and RT-qPCR was conducted using SYBR^®^ Green Pro Taq HS Premix (Accurate Biotechnology, AG11740, Changsha, China) following the manufacturer’s protocol. The thermal cycling conditions were as follows: initial denaturation at 95 °C for 30 s, followed by 40 cycles of 95 °C for 5 s and 60 °C for 30 s for annealing/extension. Relative mRNA expression levels were calculated using the 2^-ΔΔCt method, with β-actin as the internal reference gene. Primer sequences used in this study are listed in **Table 2**. In addition, to further validate the transcriptional regulation of candidate genes under inflammatory conditions *in vitro*, RT-qPCR analysis was performed in human colonic fibroblast CCD-18Co cells (GNHu59; Chinese Academy of Sciences Cell Bank). All reactions were performed in triplicate, and primer sequences for *in vitro* assays are provided in **Table 3**.

**Table 2:** The sequence of primers in q-PCR assay (tissue samples).

Gene names	Sequences
IGFBP5	F: ACCCAGTCCAAGTTTGTCGG
	R: TTGTAGAATCCTTTGCGGTCAC
β-Actin	F: TCCTTCCTGGGCATGGAGT
	R: AGCACTGTGTTGGCGTACAG

**Table 3:** The sequence of primers in q-PCR assay (*in vitro* experiments).

Gene names	Sequences
β-actin	F: GGCACCCAGCACAATGAAG
	R: CCGATCCACACGGAGTACTTG
IGFBP5	F: ACCTGAGATGAGACAGGAGTC
	R: GTAGAATCCTTTGCGGTCACAA
EGR1	F: AGCCCTACGAGCACCTGAC
	R: GGTTTGGCTGGGGTAACTG
VEGFR2	F: GGCCCAATAATCAGAGTGGCA
	R: CCAGTGTCATTTCCGATCACTTT

### 2.17 Western blot analysis

To confirm key findings at the protein level, Western blot analysis was performed on both colonic tissue and cell samples. Colonic tissues were homogenized using a mechanical homogenizer (S1873; Beyotime) in RIPA lysis buffer supplemented with phosphatase inhibitors (P0013B; Beyotime, Hangzhou, China). Cell proteins were extracted using RIPA lysis buffer containing protease and phosphatase inhibitors (Thermo Fisher Scientific). Protein concentrations were determined using a bicinchoninic acid (BCA) assay kit. Equal amounts of protein (25 μg) were separated by SDS-PAGE and transferred onto PVDF membranes (Immobilon-P, 0.45 μm; Millipore). Membranes were blocked with 5% non-fat milk in TBST (0.1% Tween-20 in Tris-buffered saline) for 1 h at room temperature. For tissue samples, membranes were incubated overnight at 4 °C with the following primary antibodies: anti-IGFBP5 (1:1000, Proteintech, China), anti-VEGFR2 (1:1000, Cell Signaling Technology, USA), anti-EGR1 (1:1000, Cell Signaling Technology, USA) and anti-ACTIN (1:1000, Boster, China, BM0627) For cell samples, the same immunoblotting procedure was applied using the following primary antibodies: anti-IGFBP5 (1:1000, Epizyme, China, Cat# P101109), anti-VEGFR2 (1:1000, Epizyme, China, Cat# P010410),anti-EGR1 (1:1000, Epizyme, China, Cat# R014802) and anti-β-actin (1:1000, Boster, China, BM0627) After three washes with TBST (10 min each), membranes were incubated with horseradish peroxidase-conjugated goat anti-rabbit secondary antibody (1:50,000, HA1001, HuaAn Bio, China) for 1 h at room temperature. Protein bands were visualized using an enhanced chemiluminescence (ECL) kit (Immobilon Western; Millipore) and imaged using a GBOX Chemi XT4 imaging system (Syngene, Cambridge, UK). Densitometric analysis was performed using GeneTools software (v4.3; Syngene), and protein expression levels were normalized to ACTIN or β-actin as appropriate. All experiments were independently repeated at least three times.

### 2.18 Enzyme-linked immunosorbent assay

To assess the inflammatory secretory phenotype of fibroblasts, culture supernatants were collected after 24 h of serum stimulation and centrifuged at 1000 × g for 10 min to remove cellular debris. The concentrations of IL-6, CCL2, and CXCL12 were determined using commercially available human ELISA kits, including Human IL-6 ELISA Kit (MultiSciences Biotech, Hangzhou, China), Human CCL2/MCP-1 ELISA Kit(MultiSciences Biotech, Hangzhou, China), and Human CXCL12/SDF-1 ELISA Kit (Wuhan Yunclone Technology Co., Ltd., Wuhan, China), according to the manufacturer’s instructions. Briefly, 100 μL of standards or samples were added to antibody-precoated microplates and incubated at 37 °C for 1.5 h, followed by incubation with biotinylated detection antibodies and streptavidin-HRP. After color development and reaction termination, absorbance was measured at 450 nm using a microplate reader. Cytokine concentrations were calculated from the corresponding standard curves. All samples were analyzed in triplicate.

### 2.19 Immunofluorescence staining

To visualize the spatial localization and co-expression of key proteins within the tissue microenvironment, immunofluorescence (IF) staining was performed on paraffin-embedded colonic tissue sections from three groups: infliximab non-responders, responders, and HC (n=9 each). Fresh tissues were fixed in 4% neutral formaldehyde overnight, dehydrated, paraffin-embedded, and sectioned at 5 μm thickness. Sections were dried at 60 °C for 1–2 h, deparaffinized in xylene, and rehydrated through graded ethanol series. Antigen retrieval was performed in citrate buffer using a microwave: high power for 5 min, then medium-low power for 15 min, followed by cooling and PBS washes. For IF staining, sections were processed and blocked as described above, then incubated at room temperature in the dark with primary antibodies against IGFBP5 (1:400, Proteintech, China) and PDGFR-α (1:200, Boster, China). Fluorescent secondary antibodies (1:200, HuaAn Bio, China) were subsequently applied for 1 h at room temperature in the dark. Nuclei were counterstained with DAPI (Beyotian, China) for 5 min before mounting. Images were captured using a Nikon Eclipse C1 fluorescence microscope. Quantification of protein expression in IF images was performed using the IF Image Analysis Toolbox plugin in ImageJ 1.54m (National Institutes of Health, USA) according to the manufacturer’s instructions (https://imagej.net/).

### 2.20 Statistical analysis

All statistical analyses were conducted using R software (v4.3.3), SPSS (v25.0), and GraphPad Prism (v9.0). Key R packages included Seurat (v5.1.0), limma (v3.58.1), sva (v3.50.0) and clusterProfiler (v4.10.1). Differential expression analyses for bulk transcriptomic, scRNA-seq, and spatial transcriptomic data were performed using the limma or Seurat pipelines with false discovery rate (FDR) correction based on the Benjamini-Hochberg method. Genes with adjusted p-values < 0.05 and |log₂FC| ≥ 1 were considered significantly differentially expressed. For GSEA analysis, significance was defined as nominal p-value < 0.05 and FDR < 0.05. Correlation analyses were performed using Spearman’s rank correlation coefficient. Experimental data are presented as mean ± standard deviation (SD). For normally distributed data with homogeneous variance, comparisons between two groups were conducted using Student’s t-test, while comparisons among multiple groups were performed using one-way ANOVA followed by appropriate *post hoc* tests. A two-sided p-value < 0.05 was considered statistically significant unless otherwise specified.

## 3 Results

### 3.1 Transcriptomic differential expression analysis in infliximab non-responders

**Figure 2:**
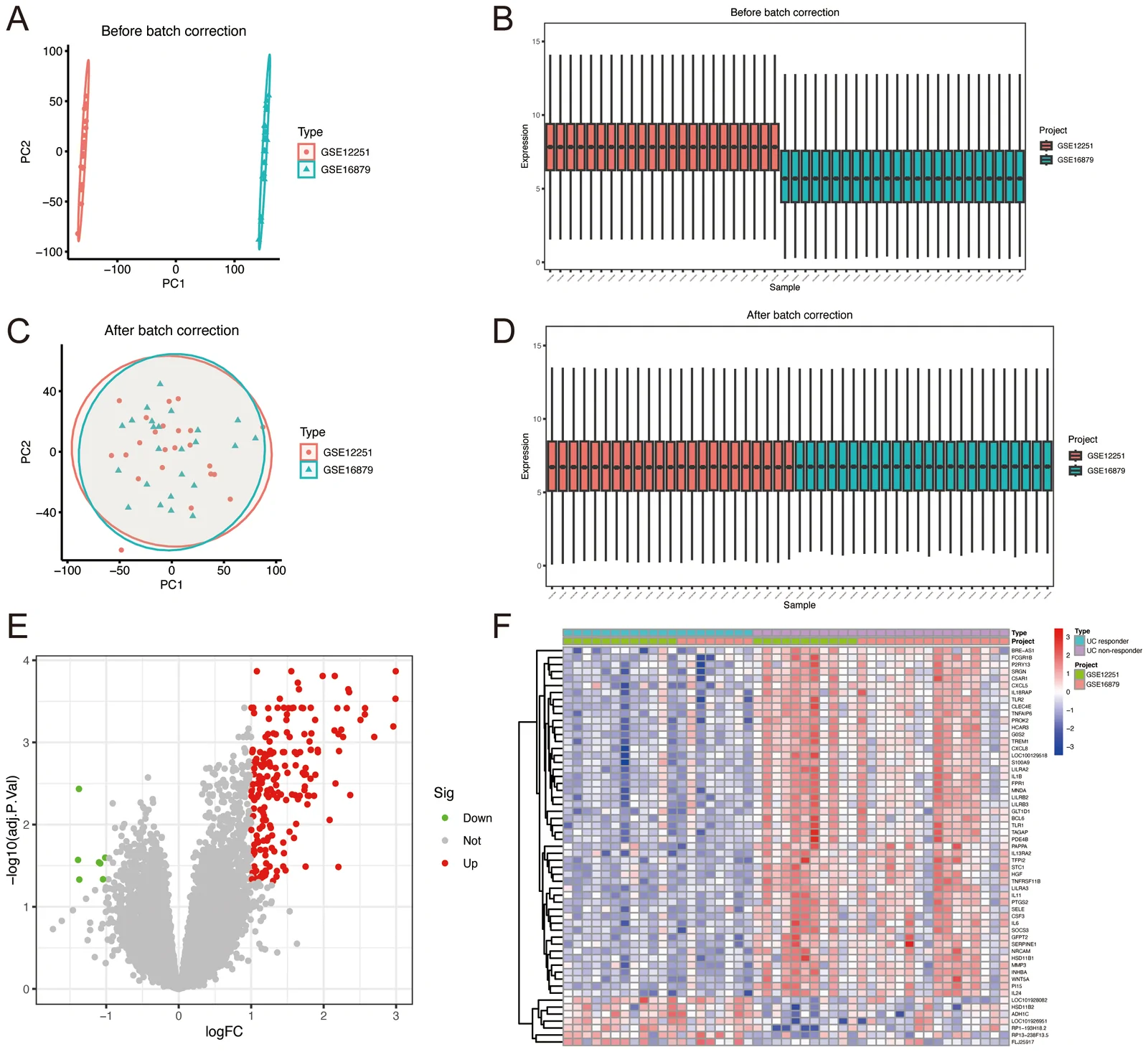
Data processing and identification of DEGs. **(A)** PCA plot prior to batch effect correction, showing distinct separation between the two GEO datasets, indicative of batch effects due to experimental sample differences. **(B)** Boxplot before batch correction, with the x-axis representing individual samples and the y-axis representing gene expression levels. Substantial variability between samples highlights the presence of batch effects. **(C)** PCA plot after batch effect correction, showing reallocation of samples and a more randomized distribution. **(D)** Boxplot post-correction, with gene expression levels aligned across all samples, reflecting successful mitigation of batch effects. **(E)** Volcano plot of DEGs in the merged dataset. **(F)** Heatmap of DEGs in the merged dataset.

The training cohort integrated 47 UC patients (27 infliximab non-responders and 20 responders) from GSE12251 and GSE16879 datasets. Post-integration batch effect correction and normalization were validated via PCA and boxplot analyses, confirming successful mitigation of inter-dataset technical variability (**Figures 2A–D**). Differential expression analysis using the limma package (|log_2_FC| ≥ 1, adjusted p-value < 0.05) identified 200 DEGs between responder phenotypes, comprising 193 upregulated and 7 downregulated genes (**Supplementary Table 2**). Top DEGs were subsequently visualized via volcano plot and heatmap analyses (**Figures 2E, F**).

### 3.2 Single-cell transcriptomic analysis of the colonic mucosal microenvironment

**Figure 3:**
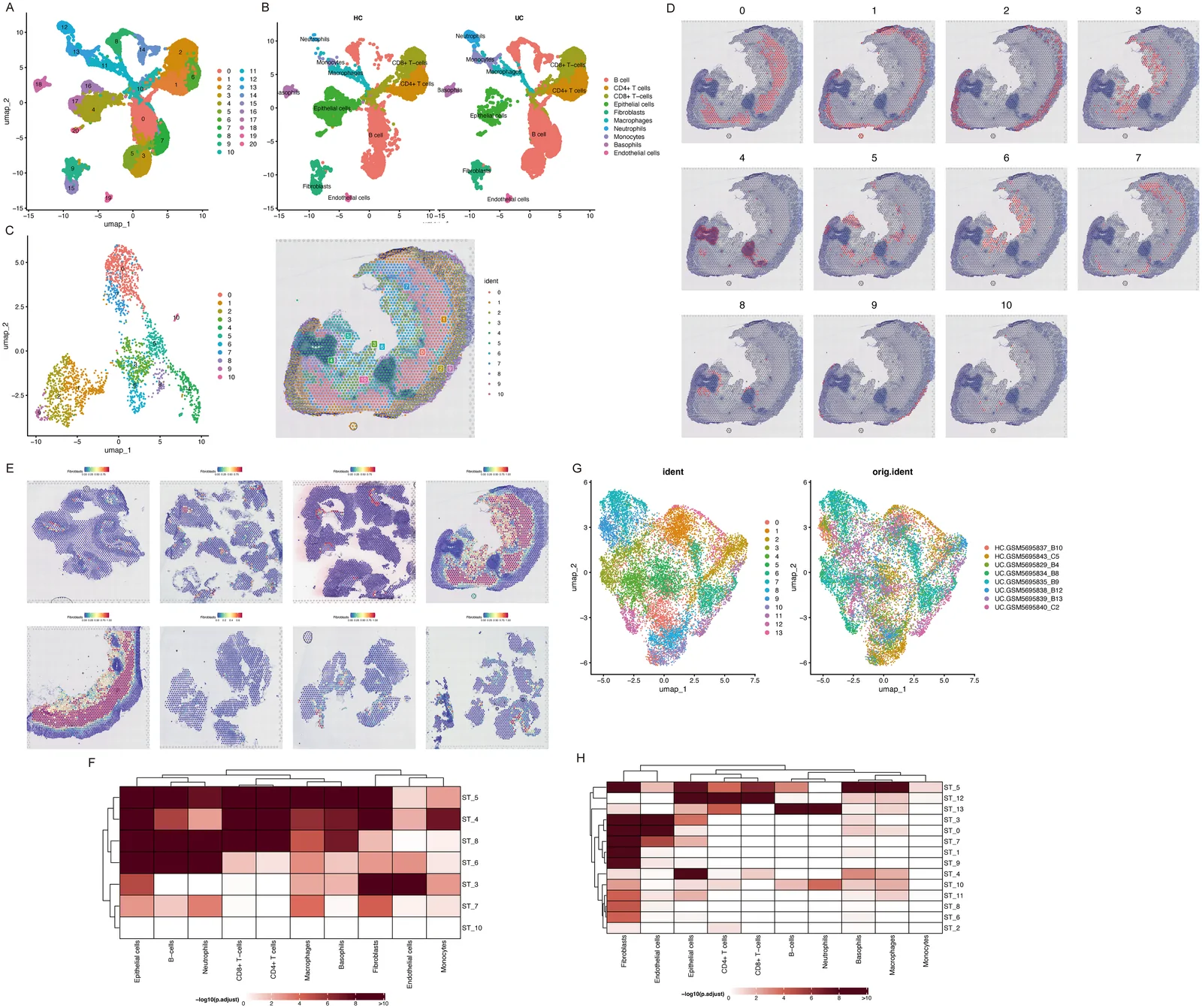
**(A)** Cellular clustering map, illustrating that distinct cell populations are assigned to different clusters. **(B)** Clustered cell classification map, with HC representing the HC group and UC representing the disease group, indicating that the various clusters correspond to their respective cell populations. **(C)** Clustering map. In the UMAP plot, each point represents a cluster, with points belonging to different clusters indicated by distinct colors. The clustering results are projected onto the corresponding spatial locations of the tissue section, where each unit in the section represents a different cluster. **(D)** Individual cluster maps. Each cluster is mapped separately onto the corresponding spatial location of the tissue section to illustrate the spatial distribution of distinct clusters. **(E)** Fibroblast mapping across all samples. The spatial distribution and enrichment of fibroblasts are shown for all samples (from left to right: control sample B10 and C5, and UC samples B4, B8, B9, B12, B13, C2). **(F)** MIA plot for UC sample B8. The horizontal axis represents distinct single-cell types, while the vertical axis represents different spatial clusters within the tissue section. Each cell in the plot indicates the degree of enrichment, with darker colors corresponding to smaller adjusted p-values and higher significance. **(G)** In the plot on the left, UMAP plot of multi-sample clustering, where points in different colors represent distinct clusters. In the plot on the right, points indicate ST samples from different sources. **(H)** Multi-sample MIA plot: the horizontal axis represents distinct single-cell types, and the vertical axis represents different spatial clusters within the tissue sections. Each cell in the plot reflects the degree of enrichment, with darker colors corresponding to smaller adjusted p-values and higher significance.

To delineate the cellular heterogeneity and composition of the colonic mucosa in UC, we performed scRNA-seq analysis on data from the GSE214695 dataset. Unsupervised clustering of cells revealed a comprehensive cellular atlas, identifying ten distinct cell populations—including B cells, CD4⁺ T cells, CD8⁺ T cells, epithelial cells, fibroblasts, macrophages, neutrophils, monocytes, basophils, and endothelial cells—which were clearly segregated in the Uniform Manifold Approximation and Projection (UMAP) space (**Figure 3A**). Comparative visualization of the clustered data between HC and UC groups further illustrated notable alterations in cellular abundance and distribution under disease conditions (**Figure 3B**). Subsequent differential expression analysis identified cell type-specific transcriptional changes in UC. Given the pivotal role of fibroblasts in tissue remodeling and inflammation, this population was selected for further evaluation of key gene expression patterns. A complete list of DEGs for fibroblasts is provided in **Supplementary Table 3**.

### 3.3 Spatial transcriptomics reveals region-specific transcriptional architectures in UC

To decipher the spatial organization of transcriptional programs in UC, we performed an integrated analysis of ST data from dataset GSE189184. This approach enabled us to resolve spatially coherent gene expression domains within the tissue architecture. As a representative case, unsupervised clustering of ST data from UC sample B8 identified ten transcriptionally distinct spatial clusters (**Figure 3C**). Projection of these cluster identities back onto the original tissue coordinates revealed a complex and heterogeneous spatial patterning, indicative of localized pathological remodeling. To delineate the precise topography of each molecular domain, we generated individual spatial distribution maps for every cluster, clearly illustrating their unique and mutually exclusive tissue occupancy (**Figure 3D**). A consistent pattern of spatially structured transcriptomic heterogeneity was observed across all analyzed samples (**Supplementary Figure 1**), confirming that UC mucosa is organized into discrete, region-specific molecular niches.

### 3.4 Multi-omics integration enables high-precision spatial mapping of cell types

To bridge single-cell resolution with spatial context, we performed an integrated analysis of scRNA-seq and ST data. This multi-omics integration strategy allows for the precise projection of cell type identities, defined at the single-cell level, onto their native spatial coordinates within the tissue architecture. We first applied this approach to map the spatial distribution of fibroblasts across all samples. The results revealed distinct and sample-specific fibroblast enrichment patterns in both HC and UC tissues (**Figure 3E**). To quantify the enrichment of various single-cell-defined populations within each spatially resolved transcriptional domain, we performed MIA. As exemplified by UC sample B8, the MIA plot demonstrates the significant enrichment of specific cell types (y-axis) within particular spatial clusters (x-axis), with the color intensity reflecting the statistical significance of enrichment (**Figure 3F**). Notably, fibroblasts were predominantly and significantly enriched in spatial clusters 3, 4, and 5 of sample B8. The MIA results for all samples are comprehensively presented in **Supplementary Figure 2**, and representative marker genes defining each spatial cluster across samples are cataloged in **Supplementary Table 4**.

### 3.5 Integrated multi-sample analysis reveals conserved spatial enrichment patterns

To systematically define the conserved and sample-shared spatial architecture in UC, we integrated all ST samples into a unified analytical framework. Unsupervised clustering of this integrated dataset revealed a consensus set of transcriptionally distinct spatial domains, visualized in a combined UMAP plot where each point represents a spot and its color denotes cluster identity (**Figure 3G**). Inter-group differential expression analysis between UC and control samples within these integrated domains identified the most significantly upregulated genes, including OLFM4, DMBT1, and IGHG3. Comprehensive lists of cluster-specific and inter-group DEGs are provided in **Supplementary Tables 5** and **6**, respectively. To decipher the cellular composition of these conserved spatial domains, we performed multi-sample MIA. The resulting enrichment heatmap (**Figure 3H**) quantitatively maps the association between single-cell-defined cell types (x-axis) and integrated spatial clusters (y-axis), with darker colors indicating higher statistical significance. This analysis confirmed a broad and significant enrichment of fibroblasts across multiple spatial clusters, most notably in clusters 0, 1, 3, 5, 7, and 9. The complete quantitative results of the multi-sample MIA are detailed in **Supplementary Table 7**.

### 3.6 Functional enrichment analysis of spatially conserved DEGs

**Figure 4:**
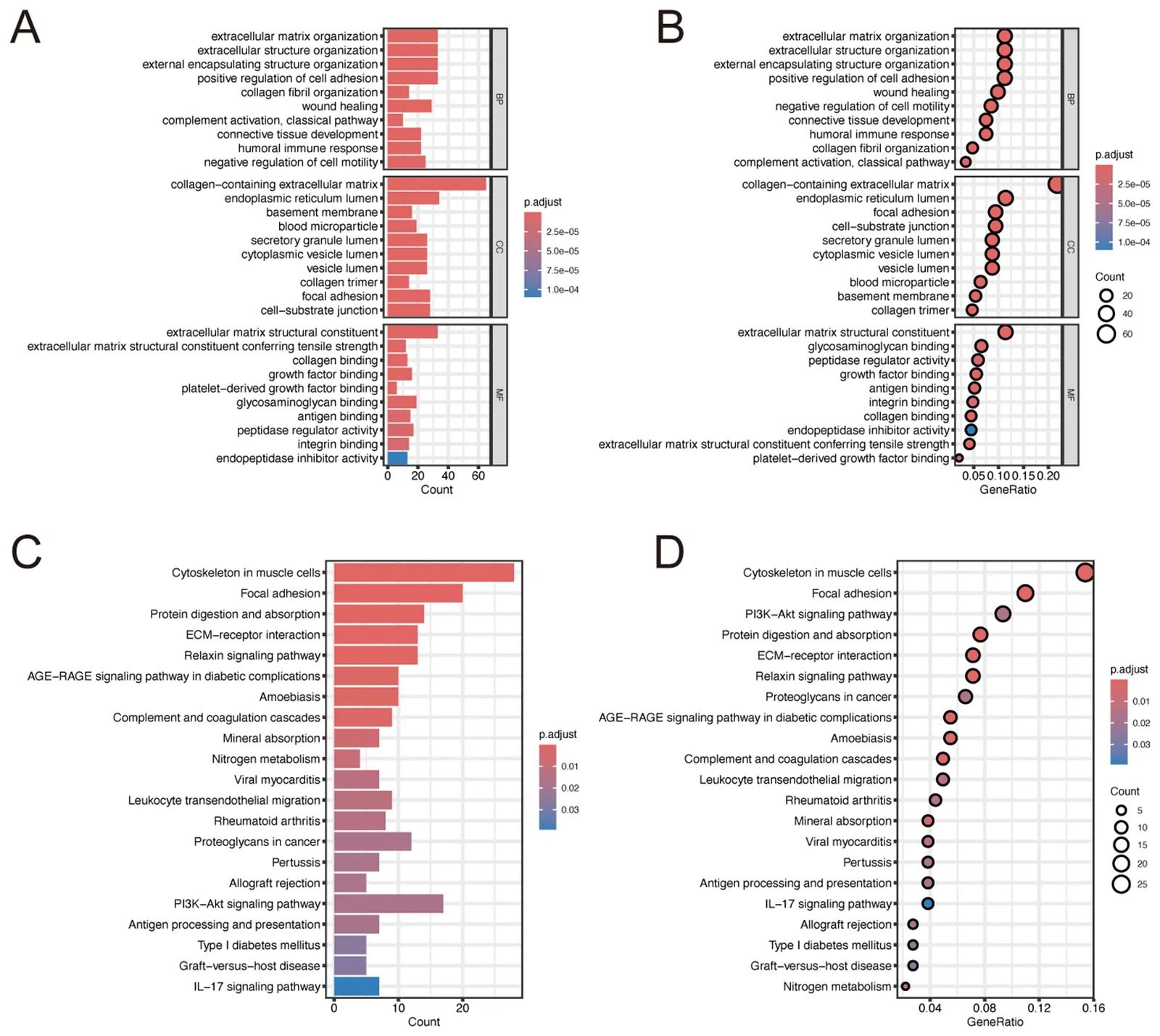
Enrichment analysis. **(A)** Bar plot of GO enrichment for intersecting genes. The x-axis represents the number of enriched genes, and the y-axis corresponds to the three GO categories: Biological Process (BP), Cellular Component (CC), and Molecular Function (MF). **(B)** Bubble plot of GO enrichment for intersecting genes. The x-axis indicates the gene ratio, and the y-axis represents the three GO categories (BP, CC, MF). Bubble size reflects the number of genes, and redder colors indicate higher significance. **(C)** Bar plot of KEGG pathway enrichment for intersecting genes. The x-axis shows the number of genes enriched in each pathway, and the y-axis lists the major enriched pathways. **(D)** Bubble plot of KEGG pathway enrichment for intersecting genes. The x-axis represents the gene ratio, and the y-axis denotes different pathways. Bubble size corresponds to the number of enriched genes, and darker colors indicate higher significance.

To elucidate the biological functions and pathways associated with the conserved spatial transcriptional alterations in UC, we performed GO and KEGG enrichment analyses on the inter-group DEGs identified from the integrated multi-sample analysis. GO analysis revealed that these genes were significantly involved in three major functional themes (**Figures 4A, B**). BP were predominantly related to extracellular matrix organization, immune response regulation, and cellular motility. CC were highly enriched in the extracellular matrix region, intracellular structures, and cell-matrix adhesion sites. MF were chiefly associated with extracellular matrix structural constituents, collagen and glycosaminoglycan binding, and growth factor receptor interactions. Concurrently, KEGG pathway analysis highlighted the involvement of these genes in several critical pathways (**Figures 4C, D**). These included: Immune-inflammatory pathways, such as the IL-17 signaling pathway, antigen processing and presentation, and leukocyte transendothelial migration; Tissue remodeling and signaling pathways, including the PI3K-Akt signaling pathway, focal adhesion, and ECM-receptor interaction; Specific disease-related pathways, exemplified by rheumatoid arthritis and the AGE-RAGE signaling pathway in diabetic complications; Metabolic processes, such as protein digestion and absorption, and mineral absorption. Collectively, these enrichment results underscore that the spatially conserved transcriptional changes in UC converge on core biological programs encompassing dysregulated immune-stromal crosstalk, altered matrix dynamics, and perturbed tissue metabolism.

### 3.7 Multi-omics integration identifies and validates core genes with fibroblast and spatial specificity

**Figure 5:**
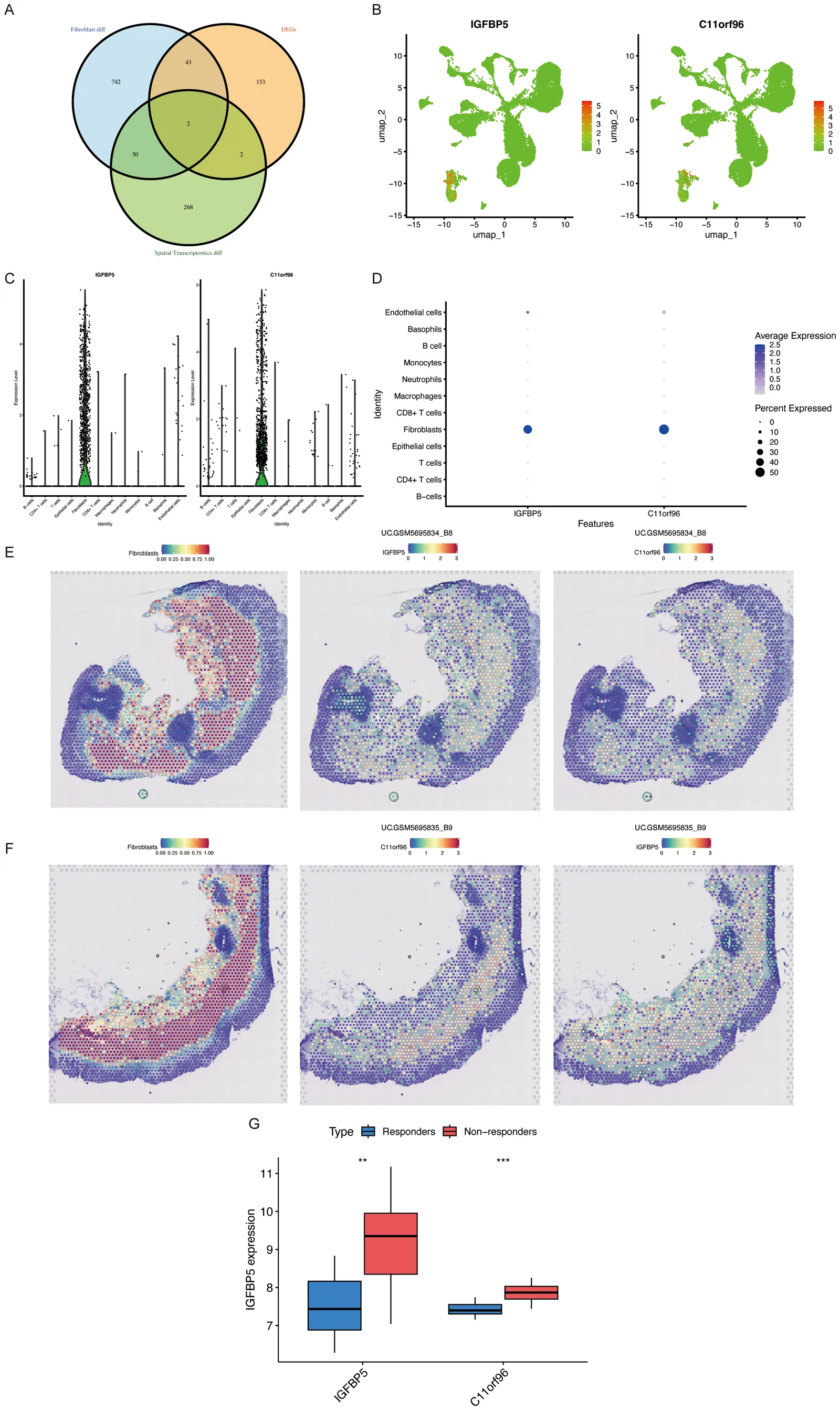
Identification and validation of core genes. **(A)** Venn diagram illustrating the intersection of three types of DEGs to identify core genes. Fibroblast diff indicates fibroblast-specific DEGs from scRNA-seq, DEGs represents intergroup DEGs from the merged transcriptomes, and ST diff denotes intergroup DEGs from the merged ST datasets.(B) Scatter plots showing the distribution and expression of core genes across different cell clusters. **(C)** Violin plots depicting the distribution and expression levels of core genes in distinct cell clusters. **(D)** Bubble plots representing the expression of core genes across cell clusters, where bubble size reflects expression intensity and color indicates expression level. **(E)** Spatial distribution of core genes in ST sample B8. **(F)** Spatial distribution of core genes in ST sample B9. **(G)** External validation of core gene expression in the independent transcriptome dataset GSE73661. (**p < 0.01, ***p < 0.001).

To pinpoint the most pivotal genes driving fibroblast-mediated pathophysiology in UC, we employed a multi-omics integration strategy. We intersected three key gene sets (1): fibroblast-specific DEGs from scRNA-seq (2), inter-group DEGs from the integrated bulk transcriptome analysis, and (3) inter-group DEGs from the merged ST datasets. This rigorous intersection identified two core candidate genes: IGFBP5 and C11orf96 (**Figure 5A**). We next characterized the expression patterns of these core genes across multiple resolutions. At the single-cell level, their expression was predominantly enriched within fibroblast clusters, as visualized through scatter plots, violin plots, and bubble plots depicting distribution and expression levels across all cell types (**Figures 5B–D**). To ascertain their spatial context, we mapped the expression of IGFBP5 and C11orf96 onto the tissue architecture. Their spatial expression domains in representative ST samples B8 and B9 showed remarkable congruence with regions previously identified as enriched for fibroblasts (**Figures 5E, F**), confirming a fibroblast-associated spatial niche. Finally, to ensure the robustness and generalizability of our findings, we validated the differential expression patterns of both core genes in an independent external transcriptomic cohort (GSE73661). The validation results strongly corroborated our internal analyses (**Figure 5G**), affirming IGFBP5 and C11orf96 as reproducible and key transcriptional features associated with UC pathology. Furthermore, IGFBP5 consistently exhibited higher expression in non-responders across three independent biologic-treated cohorts receiving vedolizumab (GSE73661), golimumab (GSE92415), and ustekinumab (GSE206285), further supporting its robust association with biologic treatment response in UC. Although C11orf96 also showed increased expression in non-responders in the vedolizumab-treated cohort, this trend was not consistently observed across the remaining validation cohorts (**Supplementary Figure 3**).

### 3.8 Core genes IGFBP5 and C11orf96 are associated with distinct immune microenvironmental features

**Figure 6:**
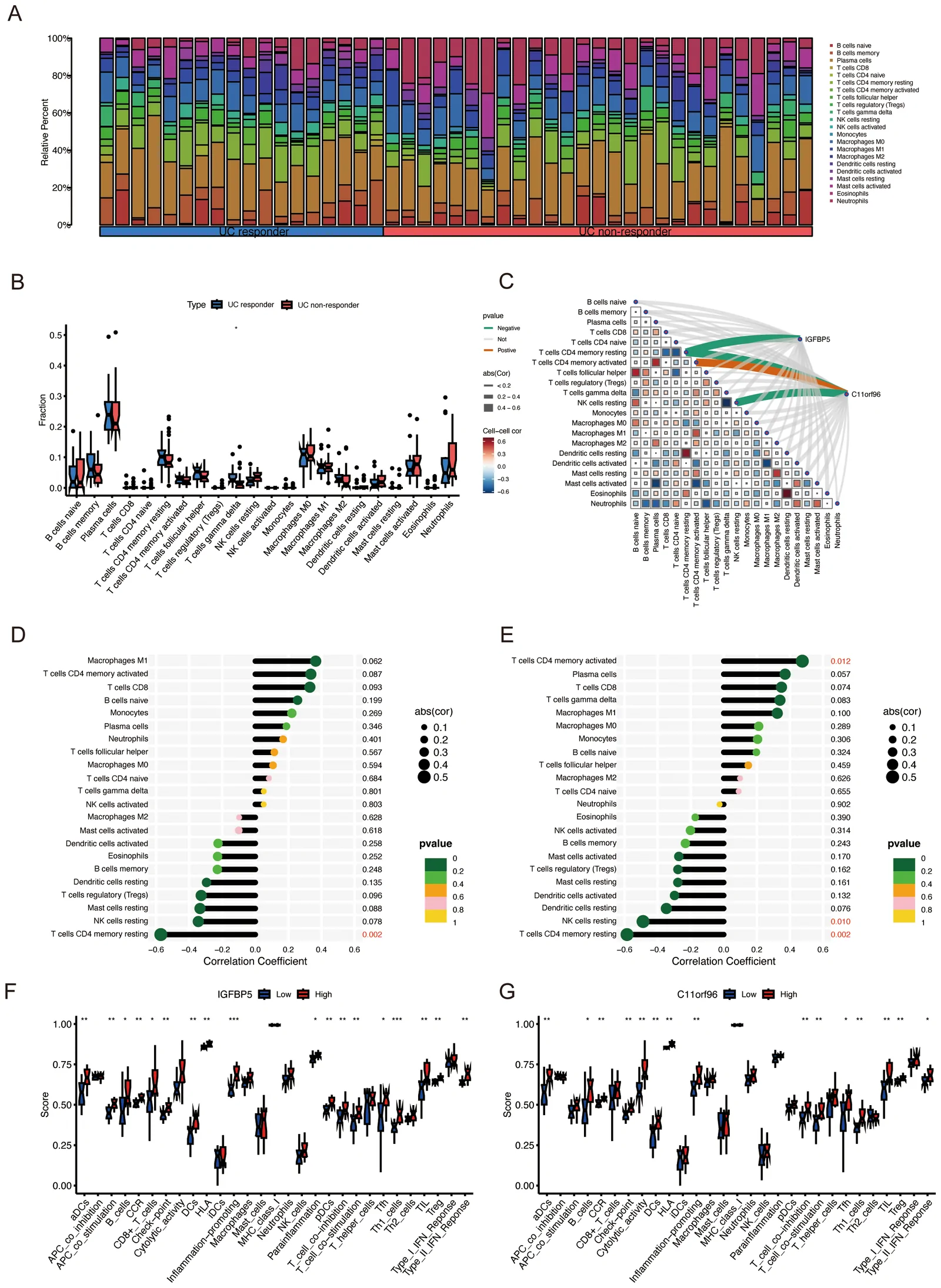
Immune cell infiltration and correlation with IGFBP5 and C11orf96. **(A)** Bar plot illustrating the relative abundance of immune cell types between UC patients who were non-responders or responders to anti-TNFα therapy (infliximab). Different colors represent distinct immune cell populations. **(B)** Violin plot highlighting significant intergroup differences in immune cell infiltration, with statistical significance indicated by asterisks (*p < 0.05, **p < 0.01, ***p < 0.001). **(C)** Heatmap depicting interactions among immune cell types. Color coding represents the degree of correlation, with gene-cell interaction lines indicating positive correlations in yellow and negative correlations in green. Line thickness corresponds to correlation strength. **(D, E)** Lollipop plots showing the correlation between core genes IGFBP5 and C11orf96 and immune cell populations, with significant associations marked by p < 0.05. **(F, G)** Violin plots illustrating immune function scores in IGFBP5 and C11orf96 high-expression groups. The x-axis represents specific immune-related functions, while the y-axis represents corresponding functional scores. Higher scores indicate greater activity of the associated immune functions, with statistical significance indicated by asterisks (*p < 0.05, **p < 0.01, ***p < 0.001).

To investigate the relationship between core gene expression and the immune microenvironment in UC patients stratified by anti-TNFα therapy response, we conducted a comprehensive immune analysis. CIBERSORT-based deconvolution of bulk transcriptomes quantified the relative abundance of 22 immune cell types between infliximab non-responders and responders (**Figure 6A**). Among the observed differences, non-responders exhibited a significantly reduced proportion of gamma delta T (γδ T) cells (**Figure 6B**). Furthermore, a correlation network analysis revealed complex interactions among various immune cell populations (**Figure 6C**). Subsequently, we examined the correlation between the two core genes, IGFBP5 and C11orf96, and immune cell infiltration. IGFBP5 expression was significantly negatively correlated with resting memory CD4⁺ T cells. In contrast, C11orf96 expression showed distinct associations, being positively correlated with activated memory CD4⁺ T cells and negatively correlated with both resting NK cells and resting memory CD4⁺ T cells (**Figures 6D, E**; detailed statistics in **Supplementary Data**). We further assessed the impact of core gene expression on global immune functional activity. Using ssGSEA, we calculated enrichment scores for a broad panel of immune-related functions and stratified patients into high- and low-expression groups based on IGFBP5 or C11orf96 levels. The high-expression group for IGFBP5 exhibited significant upregulation across multiple immune functions, most notably in pro-inflammatory response and HLA activity (**Figure 6F**). Similarly, the C11orf96 high-expression group showed enhanced activity in functions including cytolytic activity and HLA (**Figure 6G**). A substantial overlap was observed between the two groups, encompassing upregulated functions related to antigen presentation (HLA), T cell co-stimulation/co-inhibition, pro-inflammatory response, and specific lymphocyte populations (B cells, T follicular helper cells, Th1 cells). This shared signature highlights a core set of active immune processes associated with high expression of either gene.

### 3.9 GSEA and GSVA delineate distinct functional states associated with core gene expression

**Figure 7:**
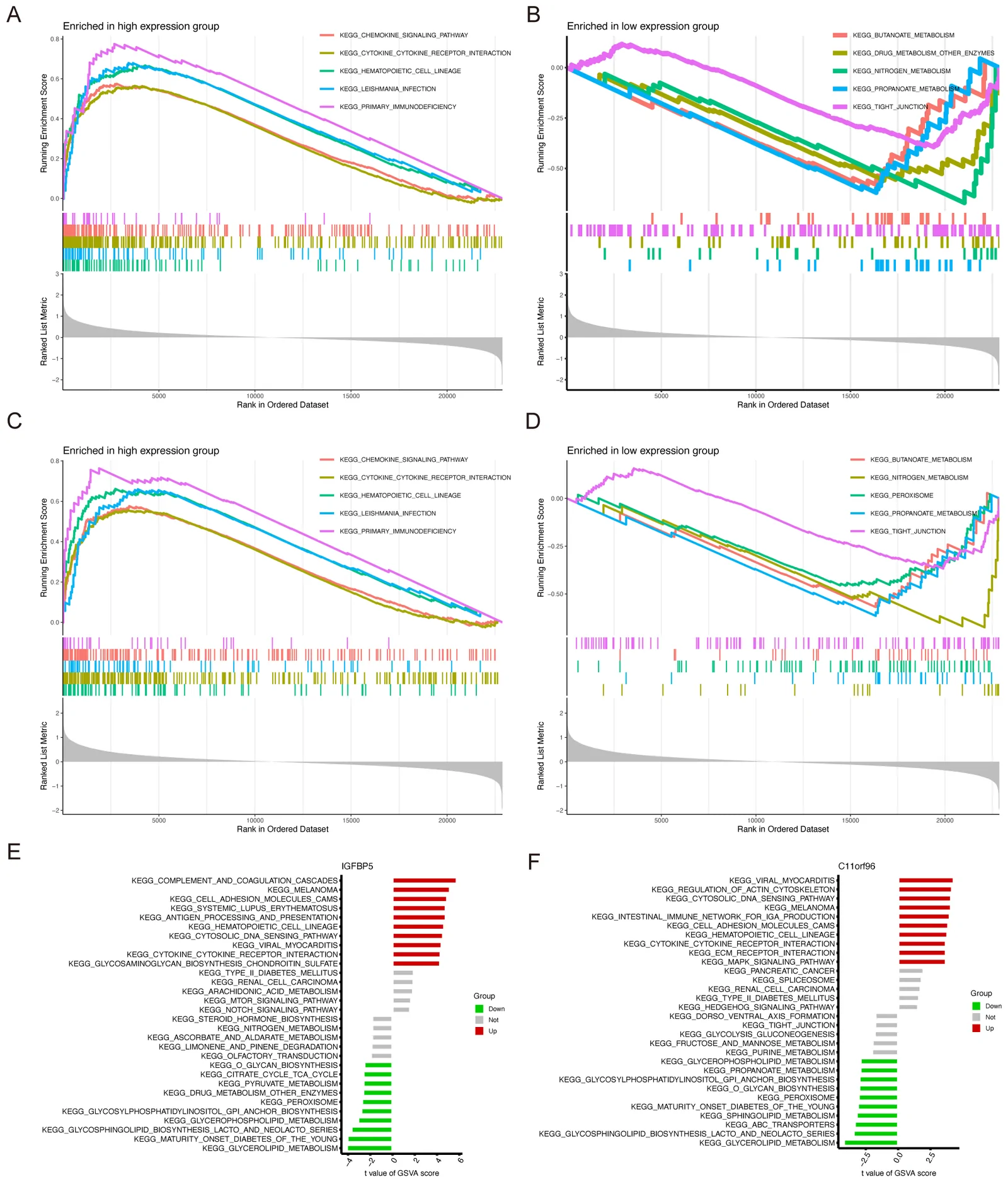
GSEA and GSVA enrichment analysis of IGFBP5 and C11orf96. **(A, B)** The top five enriched pathways in the high- and low-expression groups of IGFBP5, respectively. **(C, D)** The top five enriched pathways in the high- and low-expression groups of C11orf96, respectively. **(E, F)** Differentially enriched pathways of IGFBP5 and C11orf96, with high-expression pathways highlighted in red and low-expression pathways highlighted in green.

To characterize the global biological pathways and functional states linked to the expression of our core genes (IGFBP5 and C11orf96), we performed GSEA and GSVA. These complementary approaches revealed that high and low expression of each gene defined molecularly distinct subgroups with coherent pathway activities. GSEA indicated that high expression of both IGFBP5 and C11orf96 was associated with the enrichment of immune and inflammatory signaling pathways, such as chemokine signaling and cytokine-cytokine receptor interaction (**Figures 7A, C**). Conversely, their respective low-expression groups were predominantly enriched in metabolic processes (e.g., butanoate and nitrogen metabolism) and structural integrity pathways like tight junctions (**Figures 7B, D**). GSVA further refined this functional characterization. The IGFBP5 high-expression state was significantly associated with complement activation, antigen presentation, and specific immune-related pathways (**Figure 7E**). The C11orf96 high-expression state also showed strong immune and inflammatory links, with additional enrichment in pathways related to cytoskeletal regulation and extracellular matrix interaction (**Figure 7F**). The differential pathway enrichment patterns between high- and low-expression groups for each gene are visually summarized in the comprehensive heatmaps (**Figures 7E, F**). Collectively, these analyses demonstrate that high expression of IGFBP5 and C11orf96 demarcates a pro-inflammatory, immune-active functional state, whereas their low expression corresponds to a state oriented toward metabolic homeostasis and structural maintenance.

### 3.10 Cell communication analysis of fibroblast subpopulations stratified by IGFBP5 expression

**Figure 8:**
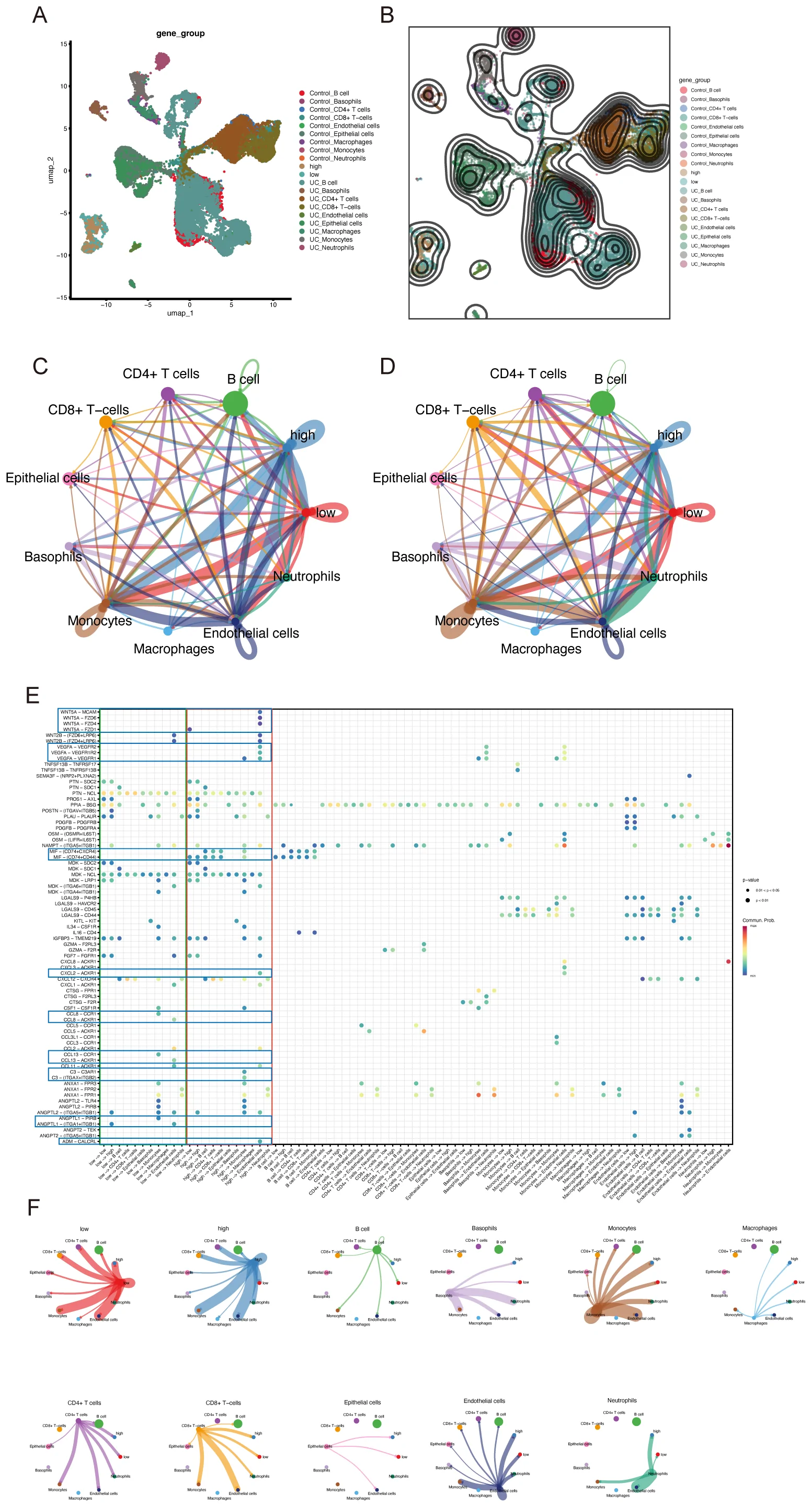
Single-cell landscape of IGFBP5⁺ and IGFBP5^-^ fibroblasts. **(A)** UMAP visualization of fibroblasts stratified into IGFBP5⁺ and IGFBP5^-^ groups. **(B)** UMAP topographic map of fibroblasts showing spatial differences between IGFBP5⁺ and IGFBP5^-^ groups. **(C)** Number of intercellular interactions between cells in IGFBP5⁺ and IGFBP5^-^ groups. **(D)** Interaction strength between cells in IGFBP5⁺ and IGFBP5^-^ groups. **(E)** Bubble plot representing intercellular communication in IGFBP5⁺ and IGFBP5^-^ groups. Bubble size reflects the number of interactions, while color intensity indicates interaction strength. **(F)** Network diagram of interactions from different cell types toward other cells, including both IGFBP5⁺ and IGFBP5^-^ fibroblasts.

Building on its association with inflammatory pathways, we investigated the role of IGFBP5 in mediating fibroblast-specific communication within the UC microenvironment. We stratified fibroblasts from our scRNA-seq dataset into IGFBP5⁺ and IGFBP5⁻ subpopulations based on expression levels. The distinct clustering and spatial distribution of these two groups within the fibroblast compartment are visualized in **Figure 8A** and B. Comparative analysis of cell-cell communication using CellChat revealed that IGFBP5⁺ fibroblasts were significantly more interactive than their IGFBP5⁻ counterparts, both in terms of the total number of interactions (**Figure 8C**) and their inferred interaction strength (**Figure 8D**). Specifically, IGFBP5⁺ fibroblasts exhibited enhanced communication with monocytes, endothelial cells, CD4⁺ T cells, and B cells. Pathway-level analysis identified the WNT, VEGF, and MIF signaling pathways as being markedly more active in the IGFBP5⁺ group (**Figure 8E**). Notably, the communication between IGFBP5⁺ fibroblasts and endothelial cells was particularly robust, involving multiple ligand-receptor pairs such as WNT5A-(FZD4/FZD6/MCAM), VEGFA-(VEGFR1/VEGFR1R2/VEGFR2), and CXCL2-ACKR1. As a signaling source, IGFBP5⁺ fibroblasts also engaged a broad network via the MIF-(CD74+CXCR4)/(CD74+CD44) axis, targeting IGFBP5⁻ fibroblasts, B cells, T cell subsets, and monocytes. The comprehensive outgoing communication network from IGFBP5⁺ fibroblasts to all other cell types, and the incoming signals they receive, are summarized in the interaction bubble plot and network diagram, respectively (**Figures 8E, F**).

### 3.11 Experimental validation of IGFBP5 expression in clinical cohorts

**Figure 9:**
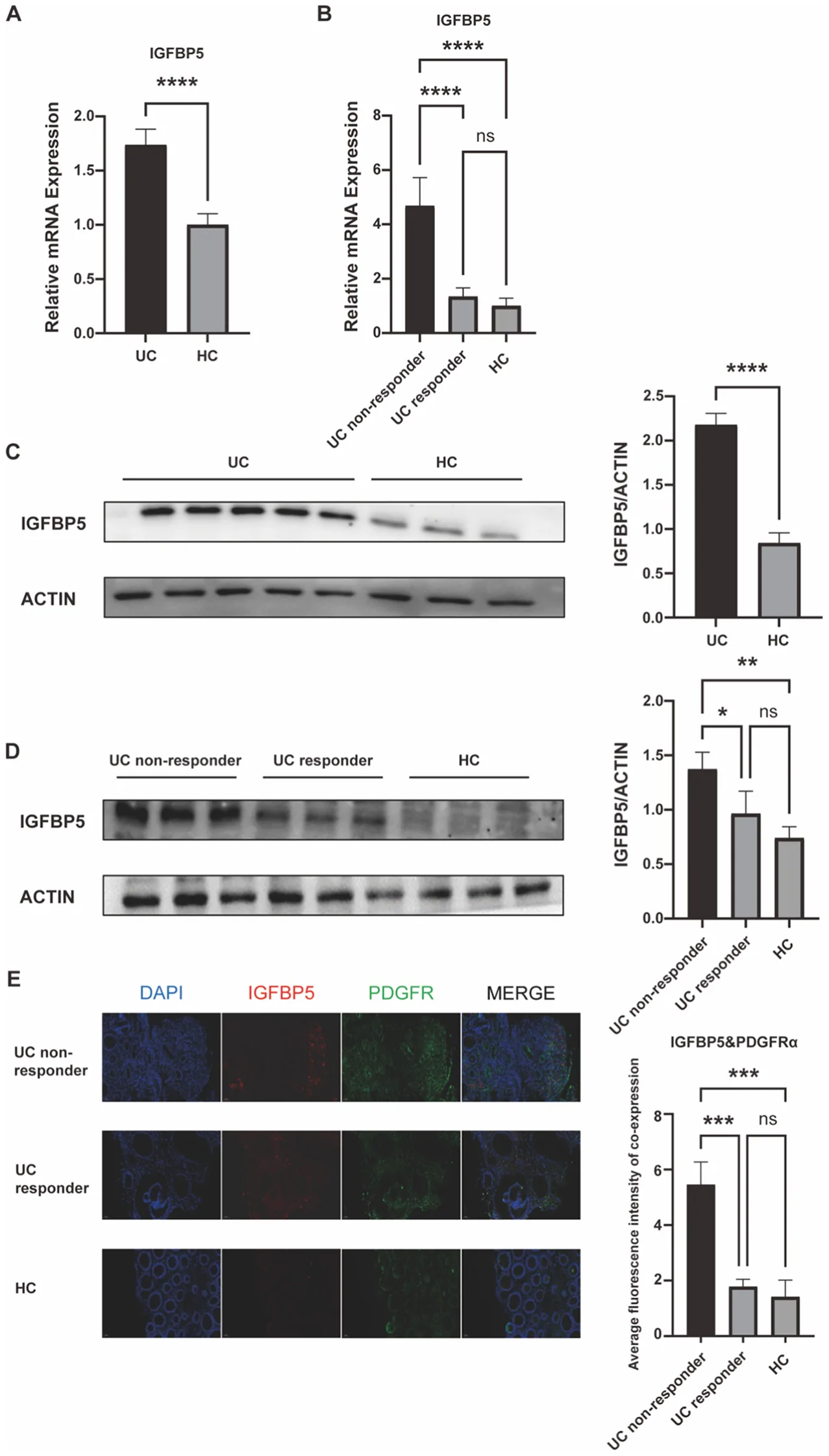
Expression of IGFBP5 in UC and HC, as well as in UC non-responders and responders, and its fluorescence co-localization with fibroblasts. **(A)** Relative mRNA expression levels of IGFBP5 in UC versus HC determined by qRT-PCR (n=9). **(B)** Relative mRNA expression levels of IGFBP5 among UC non-responders, UC responders, and HC determined by qRT-PCR (n=9). **(C)** Western blot analysis of IGFBP5 protein expression in UC versus HC and corresponding quantification (n=9). **(D)** Western blot analysis of IGFBP5 protein expression among UC non-responders, UC responders, and HC, along with quantification (n=9). **(E)** IF co-localization of IGFBP5 (green) with the fibroblast marker PDGFR-α (red) in UC non-responders, UC responders, and HC; nuclei were stained with DAPI (blue). Quantification of the average fluorescence intensity within co-expression regions is shown (n=9). Data are presented as mean ± SD. Statistical significance was assessed using one-way ANOVA and t-test. *p < 0.05, **p < 0.01, ***p < 0.001, and ****p < 0.0001; ns, not significant.

To validate our multi-omics findings at the molecular and tissue levels, we analyzed IGFBP5 expression in colonic mucosal biopsies from UC patients and HC. Quantitative RT-PCR confirmed a significant upregulation of IGFBP5 mRNA in UC tissues compared to HC (**Figure 9A**). Stratification of UC patients based on response to infliximab therapy revealed that IGFBP5 mRNA levels were highest in non-responders, significantly exceeding levels in both responders and HC (**Figure 9B**). This transcriptional upregulation was corroborated at the protein level by Western blot analysis. IGFBP5 protein expression was substantially elevated in UC tissues relative to HC (**Figure 9C**). Consistent with the mRNA data, protein abundance was markedly higher in non-responders compared to responders and HC (**Figure 9D**). To determine the cellular source of IGFBP5 within the tissue microenvironment, we performed dual IF co-staining with the fibroblast marker PDGFR-α. This analysis confirmed pronounced co-localization of IGFBP5 protein with PDGFR-α⁺ fibroblasts (**Figure 9E**). Quantitative assessment of fluorescence intensity within co-expression regions demonstrated that the signal was significantly strongest in tissues from infliximab non-responders, intermediate in responders, and lowest in HC (**Figure 9E**). These results validate the specific association of elevated IGFBP5 expression, particularly within the fibroblast compartment, with UC pathology and anti-TNFα therapy non-response.

### 3.12 The IGFBP5-EGR1-VEGFR2 axis is upregulated in infliximab non-responders

**Figure 10:**
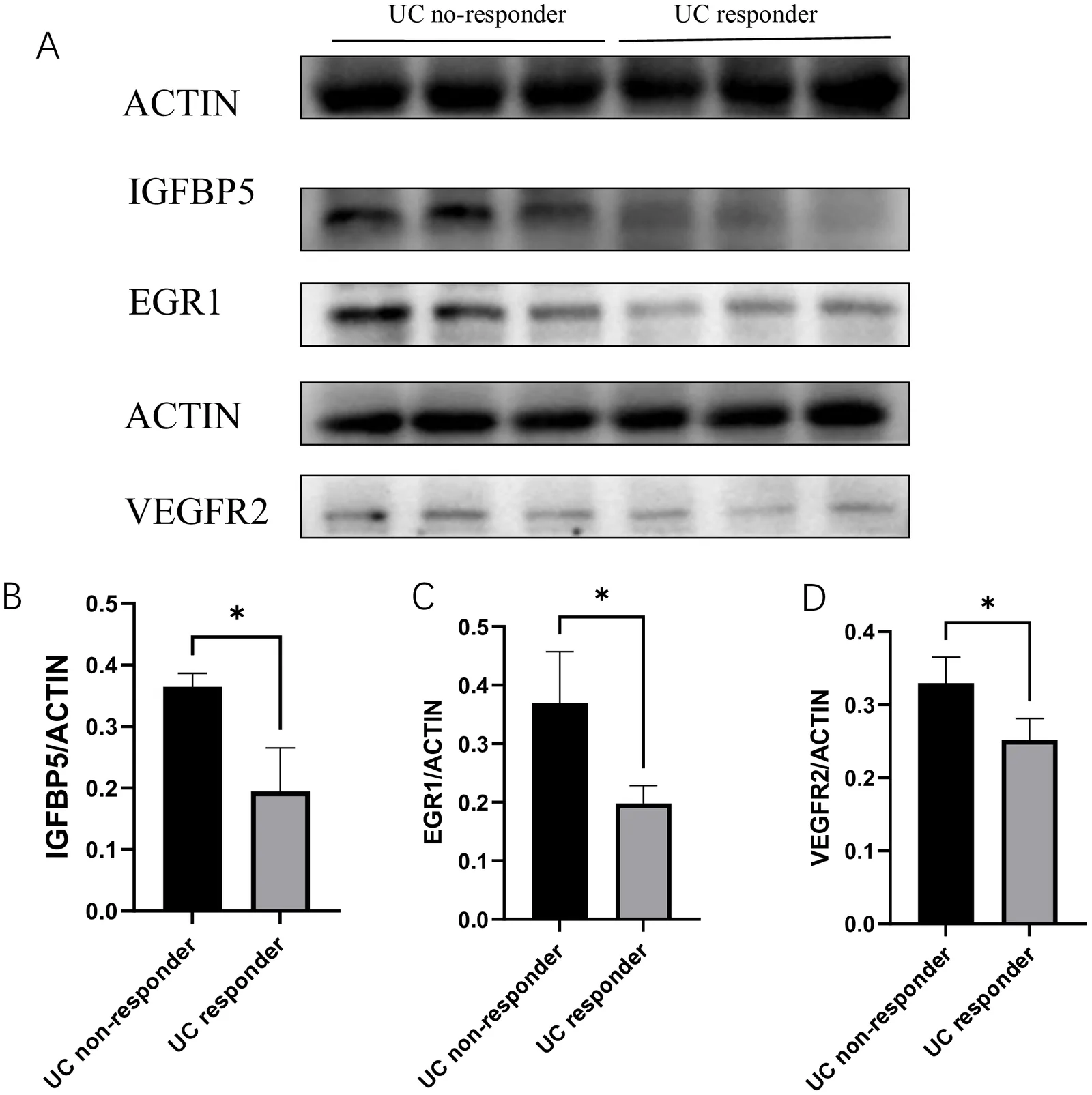
Protein Expression of the IGFBP5-EGR1-VEGFR2 Axis in colonic tissues from UC infliximab non-responders and responders. **(A)** Western blot images showing IGFBP5, EGR1, and VEGFR2 protein expression in UC non-responders and responders. **(B)** Quantification of relative IGFBP5 expression. **(C)** Quantification of relative EGR1 expression. **(D)** Quantification of relative VEGFR2 expression. *p < 0.05.

To evaluate the protein expression of IGFBP5 and its potential downstream effectors EGR1 and VEGFR2 in the context of anti-TNFα therapy resistance, we performed Western blot analysis on colonic tissues from UC infliximab non-responders and responders (R). Representative blots showed detectable protein levels for all three targets, with visibly stronger bands in the non-responder group compared to the R group (**Figure 10A**). Densitometric quantification confirmed that the protein levels of IGFBP5 (**Figure 10B**), EGR1 (**Figure 10C**), and VEGFR2 (**Figure 10D**) were all significantly elevated in the non-responder group relative to the R group. This consistent upregulation suggests a coordinated activation of the IGFBP5-EGR1-VEGFR2 axis in patients who fail to respond to infliximab therapy.

### 3.13 Activation of the IGFBP5-driven VEGFR2/EGR1 signaling axis in inflammatory colonic fibroblasts

**Figure 11:**
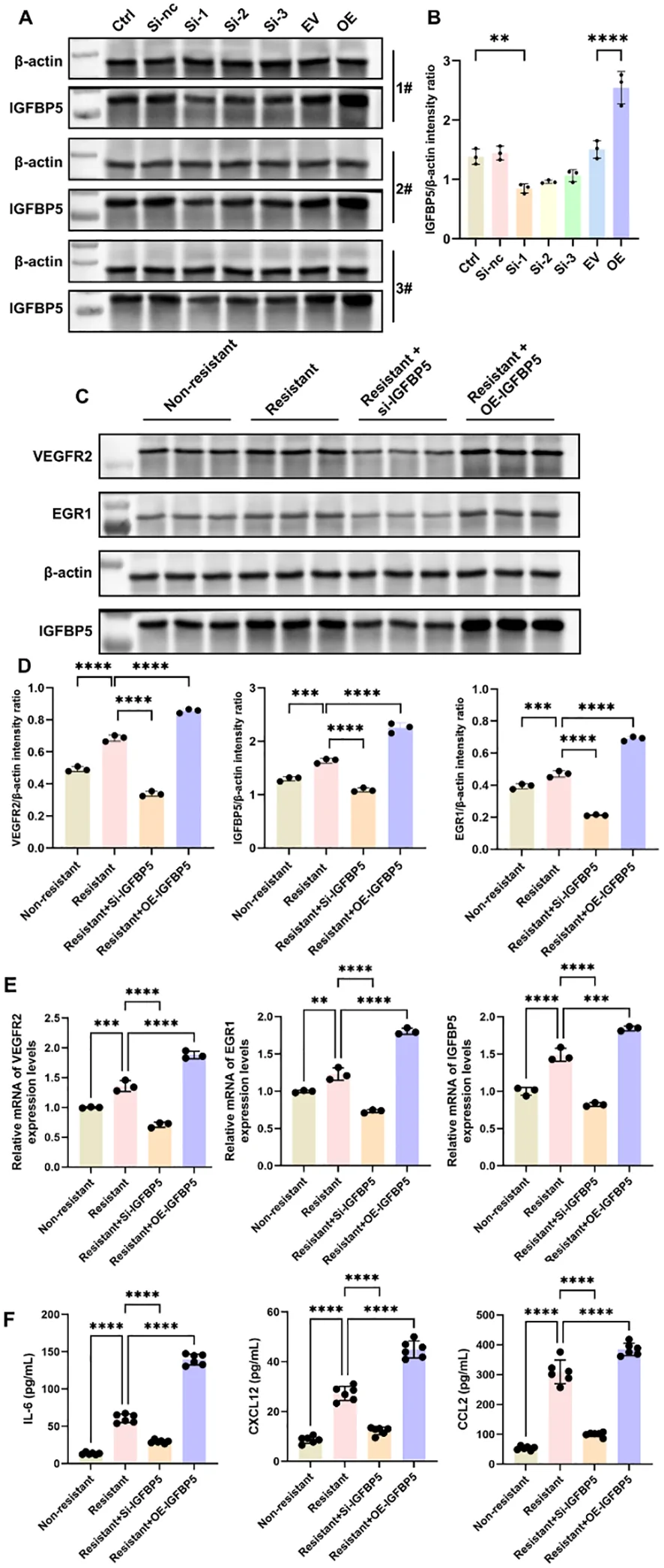
IGFBP5 regulates activation of the VEGFR2/EGR1 pathway and promotes an inflammatory fibroblast phenotype in human colonic fibroblast CCD-18Co cells exposed to serum from infliximab-resistant UC patients. **(A)** Western blot analysis of IGFBP5 knockdown and overexpression efficiency in CCD-18Co cells. Cells were transfected with negative control siRNA (si-nc), three independent IGFBP5-targeting siRNAs (si-1, si-2, and si-3), empty vector (EV), or IGFBP5 overexpression vector (OE). β-actin was used as the loading control. 1#, 2#, and 3# indicate independent experimental replicates. **(B)** Densitometric quantification of IGFBP5 protein expression, presented as the IGFBP5/β-actin intensity ratio. **(C)** Western blot analysis of VEGFR2, EGR1, and IGFBP5 protein expression in CCD-18Co cells exposed to serum from UC patients with or without infliximab resistance. Non-resistant indicates serum from UC patients without infliximab resistance, whereas Resistant indicates serum from infliximab-resistant UC patients. Under stimulation with Resistant serum, CCD-18Co cells were further subjected to IGFBP5 knockdown or IGFBP5 overexpression to evaluate downstream pathway alterations. β-actin served as the loading control. **(D)** Densitometric quantification of VEGFR2, IGFBP5, and EGR1 protein levels, expressed as VEGFR2/β-actin, IGFBP5/β-actin, and EGR1/β-actin intensity ratios, respectively. **(E)** RT-qPCR analysis of the relative mRNA expression levels of EGR1, VEGFR2, and IGFBP5 to assess IGFBP5-dependent transcriptional regulation of the VEGFR2/EGR1 pathway. **(F)** ELISA measurement of inflammatory fibroblast-associated secretory factors, including IL-6, CXCL12, and CCL2, in the cell culture supernatant. Data are presented as mean ± SD. Statistical significance is indicated in the figure: **p < 0.01, ***p < 0.001, and ****p < 0.0001.

To investigate the functional role of IGFBP5 in fibroblast activation under inflammatory conditions, CCD-18Co cells were stimulated with serum derived from infliximab-responsive and non-responsive UC patients, followed by IGFBP5 gain- and loss-of-function manipulation. Western blot analysis confirmed efficient IGFBP5 silencing and overexpression (**Figure 11A**). Among three independent siRNAs targeting IGFBP5 (si-1, si-2, and si-3), si-1 exhibited the highest knockdown efficiency and was therefore selected for subsequent gene silencing experiments (**Figures 11A, B**). Compared with serum from infliximab-responsive patients, treatment with serum from non-responsive patients significantly increased IGFBP5 expression. Concurrently, VEGFR2 and EGR1 were also upregulated under the same condition, indicating activation of the VEGFR2/EGR1 signaling axis within a drug-resistant inflammatory microenvironment. Importantly, IGFBP5 knockdown under resistant serum stimulation significantly reduced VEGFR2 and EGR1 expression. In contrast, IGFBP5 overexpression further enhanced VEGFR2 and EGR1 protein levels, exceeding those observed in the resistant serum group (**Figures 11C, D**). To determine whether IGFBP5 regulates the VEGFR2/EGR1 axis at the transcriptional level, we further quantified the mRNA expression of IGFBP5, VEGFR2, and EGR1 by RT-qPCR (**Figure 11E**). Consistent with the protein expression profiles, stimulation with serum from infliximab-non-responsive UC patients significantly increased the transcript levels of IGFBP5, VEGFR2, and EGR1 compared with serum from infliximab-responsive patients. Silencing of IGFBP5 markedly attenuated the expression of VEGFR2 and EGR1, whereas IGFBP5 overexpression further enhanced their transcriptional levels under resistant serum stimulation. These findings suggest that IGFBP5 positively regulates the VEGFR2/EGR1 signaling pathway at both the mRNA and protein levels. To evaluate whether activation of the IGFBP5-VEGFR2/EGR1 signaling axis contributes to the acquisition of an inflammatory fibroblast phenotype, we measured the secretion of representative inflammatory mediators in culture supernatants by ELISA (**Figure 11F**). Exposure to serum from infliximab-non-responsive patients significantly increased the production of IL-6, CXCL12, and CCL2 compared with serum from infliximab-responsive patients. Notably, IGFBP5 knockdown markedly reduced the secretion of these inflammatory factors, whereas IGFBP5 overexpression further promoted their release beyond the levels observed in the resistant serum group. These results indicate that elevated IGFBP5 expression enhances the pro-inflammatory activity of colonic fibroblasts and promotes the establishment of an inflammatory microenvironment through activation of the VEGFR2/EGR1 signaling cascade.

## 4 Discussion

Emerging evidence indicates that resistance to anti-TNFα therapy in inflammatory bowel disease is a multifactorial process rather than being driven solely by failure of TNF neutralization. Multiple complementary mechanisms have been proposed across recent clinical and translational studies. Pharmacokinetic variability, including anti-drug antibody formation and dynamic changes in infliximab exposure, has been associated with secondary loss of response and treatment discontinuation (17). In parallel, reduced tissue-level drug concentrations have been linked to shorter relapse-free survival, suggesting that local drug availability within inflamed mucosa may be a key determinant of therapeutic durability (18). In addition to pharmacokinetic factors, immune cell state alterations, including expansion of pathogenic Th17 cell programs, have been implicated in infliximab resistance, highlighting the contribution of adaptive immune rewiring beyond canonical TNF signaling (19). Moreover, emerging multi-omics and interspecies translation approaches have identified stromal and tissue microenvironmental pathways, such as integrin-associated signaling, as potential contributors to treatment non-response (20). Fibroblast-mediated immune interactions and dysregulated metabolic pathways have emerged as pivotal contributors to treatment resistance in UC. Collectively, these findings support a model in which anti-TNFα resistance arises from the integration of pharmacokinetic variability, immune cell reprogramming, and tissue microenvironmental remodeling (21).

In the present study, we employed an integrated multi-omics approach, combining bulk transcriptomics, scRNA-seq, and ST, to dissect the cellular and molecular underpinnings of resistance to infliximab. This strategy ultimately identified two resistance-associated candidate genes, IGFBP5 and C11orf96. Notably, UC patients non-responsive to infliximab exhibited high IGFBP5 expression alongside a pronounced enrichment of Th1 cells within the immune infiltrate, distinguishing them from responders. At the single-cell resolution, IGFBP5-positive fibroblasts demonstrated significantly enhanced intercellular communication, particularly with monocytes, endothelial cells, and CD4+ T cells, primarily via VEGF and MIF signaling pathways. These findings suggest that IGFBP5⁺ fibroblasts act as a central communication hub within the inflamed microenvironment, orchestrating both immune cell recruitment and endothelial activation. The enrichment of VEGF and MIF signaling pathways indicates a coordinated activation of angiogenic and inflammatory circuits, which may contribute to sustained immune infiltration and tissue remodeling in non-responsive UC. These insights advance our mechanistic understanding of UC resistance and highlight IGFBP5 as a potential therapeutic target. On the other hand, the observed association between IGFBP5 expression and immune activation does not imply a unidirectional regulatory relationship. Instead, IGFBP5 expression may reflect the presence of an inflammatory microenvironment while simultaneously contributing to its maintenance through fibroblast-mediated signaling. In this context, IGFBP5-positive fibroblasts may function within a dynamic feedback loop, responding to immune-derived inflammatory cues while also shaping immune cell recruitment and activation. However, the current multi-omics and computational analyses cannot fully resolve the temporal or causal directionality of these interactions, which will require further time-resolved and lineage-specific experimental validation.

The mechanisms governing fibroblast-immune cell crosstalk are being progressively elucidated and are central to therapy resistance. Interactions between fibroblasts and monocytes, for instance, play a critical role (22). Fibroblasts in the inflammatory microenvironment of UC are not a homogeneous population but instead comprise a highly heterogeneous cellular compartment with distinct functional states that differentially contribute to tissue remodeling, immune regulation, and therapeutic response. The IGFBP5-positive fibroblasts identified in this study likely represent an inflammatory functional state within this broader fibroblast lineage, sharing partial transcriptional similarities with previously described IAFs (14, 23, 24). These cells are markedly expanded in UC and are enriched for gene programs involved in fibrosis and inflammatory remodeling, including IL11, IL13RA2, and TWIST1. They also exhibit partial overlap with cancer-associated fibroblast-like transcriptional signatures, suggesting a potential role in linking chronic inflammation to tissue remodeling and a tumor-permissive microenvironment (25). Notably, these inflammation-associated fibroblasts are spatially co-localized with inflammatory myeloid populations, including monocytes and dendritic cell subsets, as well as neutrophil-enriched regions. They contribute to the formation of inflammatory cellular communication hubs through the expression of chemokines and receptors such as OSMR. The OSM-OSMR signaling axis is considered a key pathway linking myeloid cells to fibroblasts, sustaining fibroblast activation and amplifying downstream immune recruitment and inflammatory responses. This myeloid-stromal interaction network is particularly relevant in the context of anti-TNFα therapy, as adaptive immune-associated spatial structures tend to normalize following treatment, whereas inflammatory niches driven by stromal and innate immune compartments may persist and contribute to therapeutic resistance (14). TREM-1 (Triggering Receptor Expressed on Myeloid Cells 1), overexpressed on monocytes, amplifies inflammation and promotes infliximab resistance by enhancing TNFα and CCL7 production (26). Furthermore, inflammation-associated fibroblasts (IAFs) can engage in compensatory signaling with inflammatory monocytes, augmenting OSM and TNF pathways to sustain inflammation and drive resistance (14). Neutrophils, key components of the M4/M5 transcriptional signatures linked to therapy non-response in IBD (27, 28), are recruited by activated fibroblasts via IL-1β-driven, rather than TNFα-dependent, mechanisms (13). This IL-1β-dominated signature in deeply ulcerated tissue suggests a patient subset that may benefit from IL-1 pathway blockade over anti-TNFα therapy (13, 29), a concept supported by preclinical models and case studies (30–34). Hyperactivated neutrophils perpetuate inflammation via feedback loops involving IL-6, TNF-α, and chemokines, further recruiting immune cells and causing tissue damage (35). Additionally, the development of anti-drug antibodies can directly compromise anti-TNFα therapy efficacy (36).

IGFBP5, a secreted member of the insulin-like growth factor binding protein family released by multiple cell types (37), is implicated in immune regulation and autoimmunity (38). Its role in fibrosis is well-documented; in pulmonary fibrosis, activated fibroblasts overexpress IGFBP5, driving excessive production of ECM components like collagen and fibronectin (39). IGFBP5 promotes monocyte recruitment, enhances epithelial-to-mesenchymal transition (EMT) (40, 41), and preferentially induces CD4+ T cell migration, implicating it in early inflammatory and fibrotic events (42).Beyond fibrosis, IGFBP5 exerts context-dependent functions: its C-terminal domain exhibits anti-angiogenic activity in cancer by modulating Akt/ERK and NF-κB-VEGF/MMP-9 pathways (43), while in metabolic disease, serum IGFBP5 correlates with hepatic fibrosis severity (44). IGFBP5 can induce a senescence-associated, cancer-associated fibroblast (CAF)-like phenotype and promotes differentiation, fibrosis, and adhesion through multiple pathways including VEGF, NF-κB, AKT, and TGFβ (45). Notably, high IGFBP5 expression is linked to therapy resistance in cancers, enhancing resistance to agents like GNF-2 and TGX221 (46). In contrast, C11orf96 remains poorly characterized. Its gene resides on chromosome 11, a locus rich in disease-associated genes (47). The high serine content and predicted phosphorylation sites in the C11orf96 protein suggest regulation via post-translational modification (48). Its potential role in immune functions remains to be explored. However, compared with IGFBP5, the biological function and mechanistic involvement of C11orf96 in UC and anti-TNFα resistance remain largely unclear and require further investigation.

Our study reveals that IGFBP5, along with its potential effectors EGR1 and VEGFR2, is markedly upregulated in infliximab-nonresponsive UC patients, indicating that the IGFBP5-EGR1-VEGFR2 axis may be involved in anti-TNFα resistance. Moreover, functional experiments in CCD-18Co fibroblasts demonstrated that IGFBP5 knockdown reduced, whereas IGFBP5 overexpression enhanced, the expression of VEGFR2 and EGR1 at both the mRNA and protein levels. These findings provide experimental support for a regulatory relationship between IGFBP5 and the VEGFR2/EGR1 signaling pathway under inflammatory conditions. However, direct causal regulation among IGFBP5, EGR1, and VEGFR2 was not experimentally established in the current study, and the proposed IGFBP5-EGR1-VEGFR2 axis should therefore currently be considered a putative signaling network rather than a definitively established mechanistic cascade. IGFBP5, upregulated in chronic inflammatory and fibrotic diseases, is potentially involved in EGR1 via MAPK signaling to promote ECM deposition and cell migration (39). In endothelial cells, IGFBP5 enhances glycolysis through EGR1-mediated PFKFB3 upregulation, providing metabolic support for sustained inflammation (49). EGR1 itself, inducible by TNFα, exacerbates mucosal injury in IBD models by regulating MMP12 and pro-inflammatory cytokines (50). Furthermore, EGR1 directly binds to and enhances transcription of VEGFR2, a key promoter of angiogenesis (51). VEGFR2 upregulation likely sustains a local vascular network that delivers nutrients and migratory routes for inflammatory cells. Moreover, increased vascular permeability and microenvironment remodeling associated with VEGF/VEGFR2 signaling may impede anti-TNFα drug distribution in fibrotic mucosa, reducing efficacy. This aligns with observations in UC where VEGF-positive tissues correlate with elevated TNFα, IL-6, and enhanced Th1/Th17 responses (52). Thus, the IGFBP5-EGR1-VEGFR2 axis may be associated with resistance by promoting angiogenesis and microenvironment remodeling while simultaneously amplifying pro-inflammatory immune responses, thereby sustaining inflammation and undermining anti-TNFα therapy-a mechanism reminiscent of fibrosis-angiogenesis axes in other chronic pathologies like cancer (53). Consistent with this hypothesis, our ELISA analysis demonstrated that inflammatory stimulation with serum from infliximab-nonresponsive patients markedly increased the secretion of IL-6, CXCL12, and CCL2 by colonic fibroblasts, whereas IGFBP5 silencing attenuated, and IGFBP5 overexpression enhanced, the production of these mediators. IL-6 is a key amplifier of chronic intestinal inflammation and promotes pathogenic T-cell responses, while CXCL12 and CCL2 are involved in the recruitment of immune cells, including monocytes and lymphocytes, to inflamed tissues. These findings suggest that IGFBP5 may contribute to the establishment of a pro-inflammatory fibroblast phenotype and reinforce immune cell infiltration within the intestinal microenvironment. Such effects may further sustain inflammatory signaling and reduce responsiveness to anti-TNFα therapy. Interestingly, additional validation in independent cohorts treated with vedolizumab, golimumab, and ustekinumab demonstrated that IGFBP5 was consistently upregulated in non-responders across different biologic therapies. These findings suggest that IGFBP5 may serve as a predictive biomarker of treatment refractoriness rather than being restricted to infliximab or anti-TNFα-specific resistance. In contrast, C11orf96 showed significantly higher expression only in the vedolizumab-treated cohort, whereas no significant differences were observed in the golimumab- or ustekinumab-treated cohorts, indicating that its predictive value may be more therapy-dependent and requires further validation. Nevertheless, prospective studies across larger cohorts receiving different biologic therapies are still needed to establish the generalizability of IGFBP5 as a biomarker of treatment refractoriness and to clarify the therapeutic settings in which C11orf96 may have predictive value.

Our findings suggest that the IGFBP5-EGR1-VEGFR2 axis may be involved in infliximab non-response in UC, offering new prospects for mechanistic research and clinical applications. First, components of this axis could serve as candidate predictive biomarker for anti-TNFα therapy, enabling personalized treatment strategies through the early identification of patients at high risk for developing resistance. Second, targeting IGFBP5 or its downstream effectors (EGR1 and VEGFR2) therapeutically is a promising strategy. Inhibiting IGFBP5 secretion or blocking the subsequent MAPK/EGR1 pathway could attenuate fibroblast-mediated microenvironment remodeling, suppress pathological angiogenesis, and disrupt the metabolic support of inflammation. This could potentially restore sensitivity to anti-TNFα agents. Given the conserved role of this axis in fibrosis and neovascularization across chronic diseases, its relevance likely extends beyond UC. Future research should employ integrated single-cell and spatial omics to dynamically delineate the crosstalk between IGFBP5⁺ fibroblasts and immune cells and characterize associated metabolic reprogramming.

Nevertheless, several limitations of the present study should be acknowledged. Because treatment response was assessed according to the original criteria of each dataset, with different evaluation time points (4–8 weeks in the public datasets versus Week 14 in the validation cohort), some degree of heterogeneity may have been introduced into the present study. It should be acknowledged that the sample sizes of both the single-cell RNA sequencing and spatial transcriptomics datasets are relatively limited, which may restrict the statistical power and generalizability of our findings. In particular, inter-individual heterogeneity among patients with ulcerative colitis may not be fully captured in the current analysis. Although we integrated multiple independent bulk and single-cell datasets and observed consistent IGFBP5-associated patterns across different cohorts, these findings should still be interpreted with caution. Future studies involving larger, multi-center cohorts and longitudinal sampling will be essential to validate our findings, better characterize patient heterogeneity, and further clarify the role of IGFBP5-related signaling in the pathogenesis of infliximab resistance in UC. CellChat-based cell-cell communication analysis provides inferential predictions of intercellular signaling derived from transcriptional profiles. Therefore, the interactions identified in this study, including those involving IGFBP5-high fibroblasts, should be interpreted as putative communication networks rather than experimentally validated signaling events. Further functional experiments, such as ligand-receptor perturbation or co-culture assays, are required to confirm these predicted interactions. The clinical validation cohort used for qPCR, Western blot, and immunofluorescence analyses was relatively small, which may limit the statistical power and generalizability of the findings, particularly for subgroup comparisons between infliximab responders and non-responders. Moreover, immune infiltration analysis based on CIBERSORT deconvolution of bulk transcriptomic data may be affected by the substantial cellular heterogeneity of inflamed intestinal tissues. Although complementary support was provided by scRNA-seq and spatial transcriptomic analyses, strict quantitative validation using scRNA-seq-derived immune proportions or flow cytometry was not performed in the current study. Although we performed additional functional experiments in CCD-18Co fibroblasts, the current evidence remains primarily based on *in vitro* assays and patient-derived transcriptomic analyses. More complex experimental systems, including fibroblast-immune cell co-culture models, intestinal organoids, pathway-specific pharmacological inhibition studies and *in vivo* animal studies, will be required to further establish the causal role of the IGFBP5-EGR1-VEGFR2 axis in anti-TNFα resistance and to elucidate its detailed molecular mechanisms within the intestinal microenvironment. Concurrently, multi-model *in vivo* validation of the effects of modulating this axis on inflammatory progression and drug responsiveness will be crucial for translating these insights into novel therapeutic interventions. Elucidating the role of the IGFBP5-EGR1-VEGFR2 axis in immunometabolic regulation and tissue remodeling provides a refined framework for understanding UC therapy resistance and informs the development of targeted combination therapies. It should be noted that IGFBP5 upregulation may not necessarily represent a direct driver of infliximab resistance. Instead, its elevated expression in non-responders may reflect a more severe fibrotic and inflammatory microenvironment. IGFBP5 induction may also occur downstream of sustained cytokine signaling as part of a broader tissue remodeling process. Further functional and longitudinal studies are required to clarify whether IGFBP5 acts as a mediator or a biomarker of disease severity in UC. Although IGFBP5 shows a consistent association with infliximab non-response, its value as a clinically applicable predictive biomarker has not been formally validated. No ROC analysis, predictive modeling, or independent prospective cohort validation was performed in this study; therefore, its clinical utility remains preliminary. These findings should be interpreted as hypothesis-generating. Future studies with larger independent cohorts and systematic evaluation of predictive performance are required to confirm its translational potential.

## 5 Conclusion

In summary, this study identifies IGFBP5 and C11orf96 as resistance-associated candidate genes linked to infliximab non-response in UC, with subsequent multi-omics and experimental analyses primarily supporting a mechanistic role for IGFBP5 in therapy resistance. Specifically, IGFBP5⁺ fibroblasts exhibit enhanced intercellular communication with monocytes, endothelial cells, and CD4⁺ T cells, accompanied by increased activity of VEGF-related signaling pathways. Functional validation further demonstrated that IGFBP5 promotes activation of the VEGFR2/EGR1 signaling axis at both the transcriptional and protein levels and enhances the secretion of pro-inflammatory mediators, including IL-6, CXCL12, and CCL2, thereby driving the acquisition of an inflammatory fibroblast phenotype. These findings suggest that IGFBP5 may contribute to inflammatory amplification, stromal remodeling, and treatment resistance within the intestinal microenvironment of infliximab non-responders. The IGFBP5-EGR1-VEGFR2 axis may therefore serve as a potential biomarker and therapeutic target. However, further studies are required to determine whether IGFBP5 acts as a causal driver of disease pathogenesis or merely reflects disease severity in UC.

## Data Availability

The datasets presented in this study can be found in online repositories. The names of the repository/repositories and accession number(s) can be found in the article/**Supplementary Material**.
