# Supplementary material for: Multi-omics analysis identifies fibroblast IGFBP5 as a key target of anti-TNFα therapy in ulcerative colitis
Source: Front Immunol. 2026 Aug 20;17:1841149. doi: 10.3389/fimmu.2026.1841149 (PMC13540427; doi:10.3389/fimmu.2026.1841149)
Supplement: Supplementary file 1 [file DataSheet1.docx]

**Figure S1. Spatial clustering and UMAP visualization of sample cohorts in integrated spatial transcriptomics analysis.**

UMAP projections visualizing distinct transcriptional clusters. Each point represents a unique cluster defined by gene expression similarity, with point colors indicating cluster identity. Axes denote UMAP dimensions. Spatial mapping of clusters onto corresponding tissue sections. Each grid cell is colored by cluster assignment, revealing spatial distribution patterns within anatomical contexts. a-b, HC groups (B10, C5); c-g, UC groups (B4, B9, B12, B13, C2).

**Figure S2. MIA of fibroblast spatial enrichment across** **sample cohorts.**

Heatmaps showing adjusted P-values for cell type enrichment in spatial clusters. a-b, HC groups (B10, C5); c-g, UC groups (B4, B9, B12, B13, C2).

**Figure S3. Validation of IGFBP5 expression in independent biologic-treated cohorts of ulcerative colitis.**

(a) GSE73661 (vedolizumab-treated cohort). IGFBP5 and C11orf96 expression were significantly higher in non-responders than in responders (***P < 0.001). (b) GSE92415 (golimumab-treated cohort). IGFBP5 expression was significantly increased in non-responders compared with responders (**P < 0.01). (c) GSE206285 (ustekinumab-treated cohort). IGFBP5 expression was also significantly elevated in non-responders relative to responders (***P < 0.001). Statistical significance was determined using the Wilcoxon rank-sum test.
